# Health effects of kiwi wine on rats: an untargeted metabolic fingerprint study based on GC-MS/TOF†

**DOI:** 10.1039/c9ra02138h

**Published:** 2019-05-03

**Authors:** Qi Zeng, Hongjin Song, Xinyi Xu, Wenjie Mao, Hui Xie, Jimin Liang, Xueli Chen, Dan Chen, Yonghua Zhan

**Affiliations:** Engineering Research Center of Molecular and Neuro Imaging of the Ministry of Education, School of Life Science and Technology, Xidian University No. 2 South Taibai Road Xi'an Shaanxi 710071 China dchen@xidian.edu.cn yhzhan@xidian.edu.cn

## Abstract

Kiwi wine is a popular fermentation product of kiwi fruit in Asian countries. To better understand the potential health effects of kiwi wine, an untargeted gas chromatography-mass spectrometer (GC-MS) approach was taken to assess the metabolic fingerprint of rats after dietary ingestion of kiwi wine. 7 differentially expressed endogenous metabolites from serum and 8 from urine were enriched in carbohydrate metabolism, amino acid metabolism pathway, fat metabolism and other metabolisms and selected from the KEGG. The above results showed that kiwi wine mainly led to a pronounced perturbation of energy metabolism (especially carbohydrate metabolism) during the consumption period. After stopping the supply of kiwi wine 30 days later, 6 and 3 endogenous metabolites from serum and urine respectively were screened and involved in a small part of carbohydrate related amino acid metabolism and fat metabolism, which indicated that the effect of kiwi wine sustained a lasting effect on energy metabolism, amino acid metabolism and lipid metabolism after stopping the supply. Thus, kiwi wine might have a positive function on health associated with the metabolism of its constituents. To the best of our knowledge, this study provides a nutrition field view for the development of the kiwi wine agricultural industry *via* an untargeted GC-MS metabolomic approach.

## Abstract 2

*In vivo* consumption assays on rats *via* a metabonomics-based approach provide a positive view of the nutritional function of kiwi wine.

## Graphical abstract

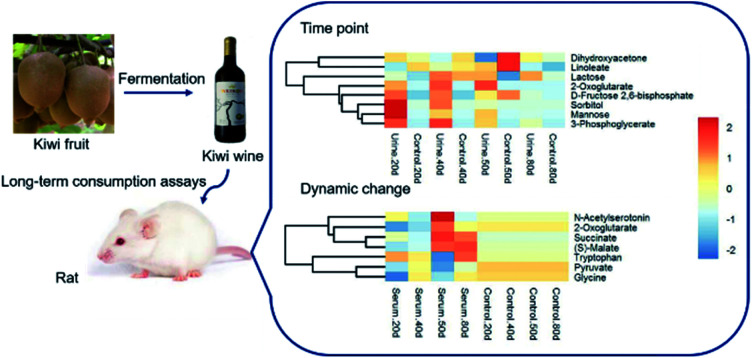

## Introduction

The kiwi fruit is well known for its health promoting effects. It has a higher concentration of vitamin C than any other commonly consumed fruits, as well as high levels of fibre, vitamins E and K, folate, carotenoids, flavonoids and polyphenols.^1^ In addition, many studies have provided evidence on the beneficial effects of the kiwi fruit, including laxation activity,^2^ regulating adipocyte differentiation and function,^3^ anti-inflammatory property,^4^ cardiovascular protective properties,^5^ antimicrobial activity^6^ and antioxidant activity.^7^ However, storage of the fresh fruit is a main challenge for the whole industry. Therefore, derivative products have been developed and these are initially valued for their improved shelf life, safety and organoleptic properties.^8^

As a result of recent discoveries on the benefits of multitude food–microbe combinations, fermented foods have regained popularity due to their potential health-promoting properties.^8^ And moreover, fermented foods were considered as part of dietary supplementation.^9,10^ In a sense, wine is one of the simple fermentation products of fruits or grains, and has been shown by numerous studies to possess beneficial properties when consumed under moderation.^11^ However, most researches are focused on the health effects of red wine (from grapes), while other fruit wines have rarely been evaluated. In Asian countries and especially in China, kiwi has been used for several decades to produce wine.^12^ Related studies mainly focus on the fermentation conditions, quality evaluation, and analysis of its contents.^12–14^ However, few studies have investigated the mechanism by which kiwi wine exerts its beneficial effects on health.

Nowadays, metabolomics screening is considered a powerful tool for identifying biomarkers and is commonly used for the assessment of functional foods.^15^ Seemingly random metabolite information could offer information on the complex relationship between diet and health through in-depth data exploration. For example, a series of untargeted metabolomics studies have been performed to evaluate the health impact of grape derivatives through nuclear magnetic resonance (NMR) technology, gas chromatography-mass spectrometer (GC-MS), and high-performance liquid chromatography-mass spectrometry (LC-MS) approaches.^16–19^ GC-MS has been proved to be a robust, unbiased method for identifying and quantifying metabolites with high sensitivity, reproducibility, simplicity, and easily accessible to the National Institute of Standards and Technology (NIST) database.^20^ However, so far, no metabolomics approach studies have been undertaken to evaluate the impact of kiwi wine consumption. Therefore, the gas chromatography-mass spectrometry/time of flight (GC-MS/TOF) method might be potentially the most suitable choice for the primary analysis of kiwi wine, considering the integrity and reliability of this approach.

**Fig. 1:**
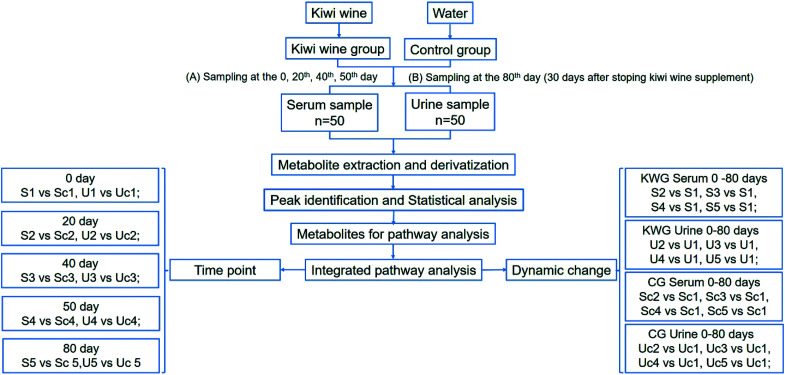
Total design of the experiment. The experiment was taken on rats, which were undivided into two groups (kiwi wine, KWG and control group, CG). The serum and urine samples were collected at the 0, 20^th^, 40^th^, 50^th^ days and one month (30 days) later after stopping kiwi wine supply. Metabolites were analyzed by GC-MS/TOF and identified by comparing with database. Data were deeper dig for screened significant metabolites and relative pathways from two aspects (time point and dynamic change) based on statistical model.

In this study, we focused on kiwi wine produced in Zhouzhi county (Shaanxi Province, China), which is produced by fermentation by the yeast *Saccharomyces cerevisiae* for two years and with the alcohol content of 11.8%. Our previous analysis revealed that it contains 117 compounds (Table S1†). Among these compounds, sugars were main constituents of the kiwi wine. However, excessive sugar consumption has been linked to several metabolic abnormalities and adverse health conditions.^21^ At the same time, fermentation products are considered beneficial for health.^22^ Therefore, in order to investigate the potential kiwi wine dietary supplementation effects on health, we performed long-term *in vivo* kiwi wine consumption assays on rats. Meanwhile, the metabolic changes were evaluated in serum and urine samples by GC-MS/TOF analysis and analyzed by both time point analysis models and dynamic change analysis model (Fig. 1).

## Materials and methods

### Chemicals and diets

Kiwi wine was obtained from the Kathy Dainy Wine Company (strain: *Saccharomyces cerevisiae*, ATCC 9763; Kathy Dainy Wine Co., LTD, Shaanxi, China). Rats were purchased from the Xian Jiaotong University Health Science Center, China. The internal standard (l-2-chlorophenylalanine, CAS#: 103616-89-3, ≥98%) was purchased from the Shanghai Hengbai Biotech Co. Ltd (Shanghai, China). The derivatization reagent BSTFA was purchased from REGIS Technologies. Inc. (Morton Grove, USA). Other chemicals and reagents were obtained from Sigma-Aldrich Chemical Co. Ltd. (St. Louis, MO, USA), unless otherwise stated.

### Animals and treatment

Female SD rats (4–6 weeks old) were obtained from the Xian Jiaotong University Health Science Center, China, and housed under a specific-pathogen-free (SPF) conditions at 25 ± 2 °C, 50–60% humidity, and a light/dark cycle of 12 hours. Animals had ad libitum access to water and standard laboratory animal diet. 8 weeks after social maturity, the adult rats were randomly grouped into two groups as the kiwi wine group (KWG) and the control group (CG) and treated with kiwi wine (6 repeats) and water (4 repeats), respectively. There was no significant weight difference between KWG and CG as the *P* value was 0.2622. The administration dose of kiwi wine was calculated according to the health standards of grape wine. According to the Wine & Spirit Education Trust (WSET) guidelines, the alcohol consumption for each person should not exceed 4 units per day and the healthy standard for men is 3 units, and 2 units for females (1 unit = 12 mL). The alcohol content of kiwi wine is 11.8%, so the equivalent amount for rats was estimated to be 3.35 mL per kg per day. To investigate the effects of kiwi wine the rats were daily administered with wine *via* an oral gavage at the same time of the day. During the experiment, the body weights of rats were recorded every three days. The whole experiment lasted for 80 days. All animal procedures were performed in accordance with the internationally accepted laboratory animal use and care guidelines^23^ and the protocol was approved by the Xian Jiaotong University Animal Care and Use Committee (number XJTULAC2016-412).

### Sample preparation

During the experiment period, serum and urine samples were collected at the 0, 20th, 40th and 50th day of treatment. Then kiwi wine administration was stopped and 30 days later, final samples of blood and urine were collected, before the sacrifice of rats (sample abbreviations are listed in Table 1). Food and water supplementation were ceased 12 hours before sampling. Blood samples (1.5 mL from each rat) were collected from the tail vein. After 40 minutes standing, the blood samples were centrifuged at 2000 rpm for 5 minutes. Next, the supernatant was centrifuged for 5 minutes and the serum sample was collected. The collected serum samples (>200 μL) were placed into 2 mL EP tubes and stored at −80 °C until further analysis. The collected urine samples were also stored at −80 °C until further analysis. The wine sample was stored at 4 °C prior to analysis.

**Table 1:** The information of sample groups

Time of sampling	Kiwi wine group (KWG, 6 repeat)		Control group (CG, 4 repeat)	
	Serum	Urine	Serum	Urine
0	S1	U1	Sc1	Uc1
20	S2	U2	Sc2	Uc2
40	S3	U3	Sc3	Uc3
50	S4	U4	Sc4	Uc4
1 M later	S5	U5	Sc5	Uc5

### Sample preparation for GC-MS analysis

For serum analysis, 20 μL of l-2-chlorophenylalanine (1 mg mL^−1^ stock in dH_2_O), as an internal standard, and 0.35 mL of methanol were added to 100 μL of serum samples in 1.5 mL EP tubes. The mixture was vortexed for 10 s, then centrifuged for 15 minutes at 13 000 rpm, at 4 °C, and the supernatant (0.39 mL) was transferred into a fresh 2 mL GC/MS glass vial. 8 μL of each sample was pooled to prepare the quality control (QC) sample. For urine analysis, 20 μL of urease (80 mg mL^−1^ in water) were added to 100 μL of urine sample in 1.5 mL EP tubes, and the sample was vortexed for 30 seconds before incubation at 37 °C for 1 hour. 0.35 mL of methanol and 20 μL of l-2-chlorophenylalanine (1 mg mL^−1^ stock in dH_2_O), as an internal standard, we added and mixed by vortexing for 30 seconds. Then the samples were centrifuged for 15 min at 13 000 rpm, at 4 °C. The supernatant (0.4 mL) was transferred into a fresh 2 mL GC/MS glass vial, and 8 μL of each sample was pooled as the QC sample. Following, derivatization reactions were performed to modify the urine samples. All the samples were dried in a vacuum centrifuge without heating.

Derivatization reactions were performed to modify the serum samples. Samples were dried in an air vacuum centrifuge without heating. 60 μL (serum) or 80 μL (urine) of methoxy amination hydrochloride (20 mg mL^−1^ in pyridine) was added and the samples were incubated for 30 minutes at 80 °C. Following, 80 μL (serum) or 100 μL (urine) of BSTFA reagent (1% TMCS, v/v) was added to each sample, and all the samples were incubated for 1.5 hours at 70 °C. Finally, 8 μL (serum) or 10 μL (urine) of FAMEs (standard mixture of fatty acid methyl esters; C8–C16: 1 mg mL; C18–C24: 0.5 mg mL^−1^, in chloroform) were added to the QC sample after cooling the sample at room temperature. All samples were well mixed prior to GC-MS analysis.

### GC-MS analysis

GC-MS/TOF analysis was performed using the Agilent 7890 gas chromatograph system coupled with a Pegasus HT time-of-flight mass spectrometer. The system utilized a DB-5MS capillary column coated with 5% diphenyl cross-linked with 95% dimethylpolysiloxane (30 m × 250 μm inner diameter, 0.25 μm film thickness; J&W Scientific, Folsom, CA, USA).

1 μL (serum) and 2 μL (urine) aliquots were injected through a vaporizing injector in the splitless mode. Helium was used as the carrier gas; the front inlet purge flow was set at 3 mL min^−1^, and the gas flow rate through the column was set at 1 mL min^−1^. The initial temperature was kept at 50 °C for 1 min, then raised to 310 °C at a rate of 20 °C min^−1^ (serum) and 10 °C min^−1^ (urine), and finally kept for 6 min (serum) and 3 min (urine) at 310 °C. The injection, the transfer line, and the ion source temperatures were set at 280, 270, and 220 °C, respectively. The energy was −70 eV in electron impact mode. The mass spectrometry data were acquired in full-scan mode with the *m*/*z* range of 30–600 (serum) and 50–500 (urine) at a rate of 20 spectra per second after a solvent delay of 366 seconds (serum) and 455 seconds (urine).

### Metabolomics data analysis

Chroma TOF 4.3X software of LECO Corporation and LECO-Fiehn Rtx5 database were used for systematical extraction of raw peaks, baseline, the data from baseline filtering, peak alignment, deconvolution analysis, peak identification and integration of the peak area.^24^ The RI (retention time index) method was used for peak identification, and the RI tolerance was 5000. Remove metabolic features were detected in <50% of the QC samples.^25^

Metabolites were identified through the interquartile range de-noising method.^25^ The missing values of the raw data were filled with half of the minimum value. In addition, internal standard normalization method was employed in this data analysis. The resulted three-dimensional data, including the peak number, sample name, and normalized peak area, were incorporated into the SIMCA14.1 software package (V14.1, MKS Data Analytics Solutions, Umea, Sweden) for Principal Component Analysis (PCA) and Orthogonal Projections to Latent Structures-Discriminate Analysis (OPLS-DA). PCA shows the distribution of the original data.^26^ In order to obtain a higher level of group separation and get a better understanding of variables responsible for classification, OPLS-DA was applied, while the OPLS-DA model was tested by the permutation test (*n* = 200).^27^ 7-fold cross validation was used to estimate the robustness and the predictability of the model; the model was validated by the permutation test. Based on the OPLS-DA, a loading plot was constructed, which showed the contribution of variables to differences between the two groups. It also showed the important variables which were situated far from the origin, but the loading plot was complex due to the incorporation of many variables. To refine this analysis, the first principal component of Variable Importance in the Projection (VIP) was obtained. In test; variables were discarded between two comparison groups. In addition, public databases including KEGG (http://www.genome.jp/kegg/) and NIST (http://www.nist.gov/index.html) were utilized to search for contributing pathways of metabolites.^24,28^ In addition, the freely step 2, the remaining variables were assessed by Student's *t*-available web-based tool, MetaboAnalyst (http://www.metaboanalyst.ca), which incorporates the high-quality KEGG metabolic pathway data, was used for pathway analysis.

## Results

### Effect of kiwi wine on the body weight

**Fig. 2:**
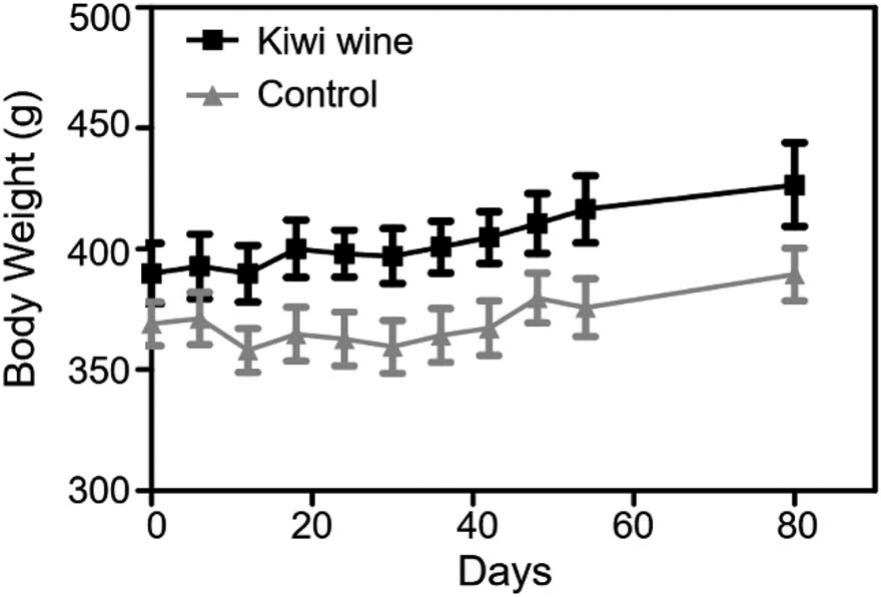
Body weight of controls and kiwi wine rats for 80 days. The body weights of rats were recorded every three days. The whole experiment lasted for 80 days. And before the experiment, there was no significant different between Kiwi wine group and control group as the *p* value = 0.2622.

Throughout the animal experiment, there were no obvious differences in common food and water consumption among the two groups. During the 90 days treatment, rats fed with kiwi wine had a stable weight fluctuation compared to those fed with water (Fig. 2). The increase of body weight in the KWG and the CG was 6.85%, and 2.65%, respectively, during the first experimental period. However, in the second experimental period (after stopping kiwi wine for 30 days) the body weight increase was stabilized at 2.35% for the KWG and 3.73% for the CG. During the kiwi wine consumption, the KWG showed a higher increase in body weight relative to the CG; while after stopping kiwi wine supplementation, the increase trend of body weight significantly declined.

### Data acquisition quality

Typical ion chromatograms (TIC) are displayed in ESI Fig. S1.† In the principal component analysis score plots of both the discovery and test sets (ESI Fig. S2†), the QC samples clustered tightly together, which confirmed the reliability of the present study. Additionally, correlation of the QC samples showed that the present analysis was stable and repeatable (ESI Fig. S3†).

**Fig. 3:**
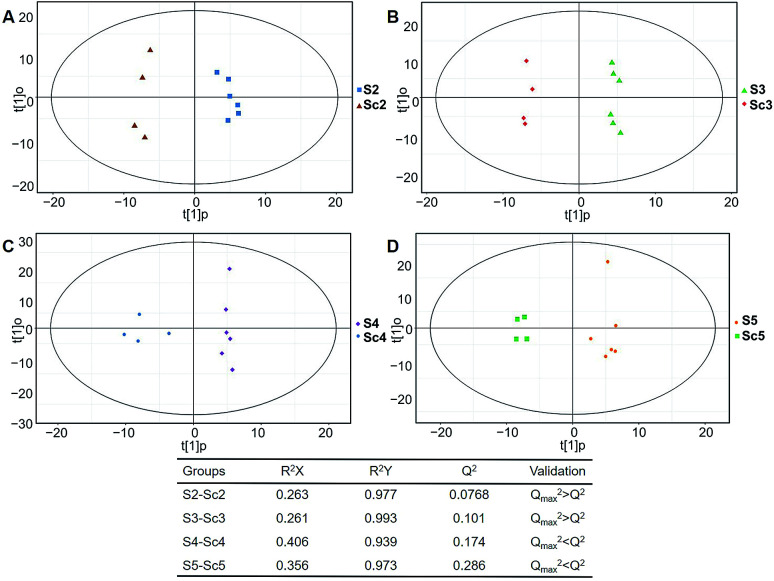
Plots of OPLS-DA and validation of the OPLS-Da model (serum). (A) Day 20 KWG (S2) *vs.* CG (Sc2); (B) day 40 KWG (S3) *vs.* CG (Sc3); (C) day 50 KWG (S4) *vs.* CG (Sc4); (D) day 80 KWG (S5) *vs.* CG (Sc5). *R*^2^*X* and *R*^2^*Y* are the cumulative modelled variation in the *X* and *Y* matrix, respectively; *Q*^2^ is the cumulative predicted variation in the *Y* matrix; validation: 7-fold cross validation was used to estimate the robustness and the predictive ability of our model, such permutation test was proceeded in order to further validate the model. After 200 permutations, the low values of *Q*^2^ (*Q*_max_^2^ > *Q*^2^) intercept indicate the robustness of the models, and thus show a low risk of over fitting and reliable.

**Fig. 4:**
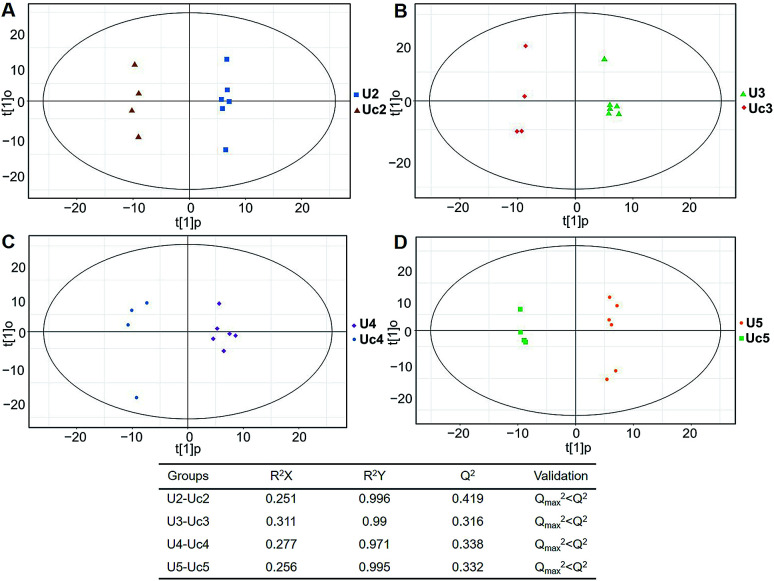
Plots of OPLS-DA and validation of the OPLS-Da model (urine). (A) Day 20 KWG (U2) *vs.* CG (Uc2); (B) day 40 KWG (U3) *vs.* CG (Uc3); (C) day 50 KWG (U4) *vs.* CG (Uc4); (D) day 80 KWG (U5) *vs.* CG (Uc5). *R*^2^*X* and *R*^2^*Y* are the cumulative modelled variation in the *X* and *Y* matrix, respectively; *Q*^2^ is the cumulative predicted variation in the *Y* matrix; the validation was same with the description in Fig. 3.

**Fig. 5:**
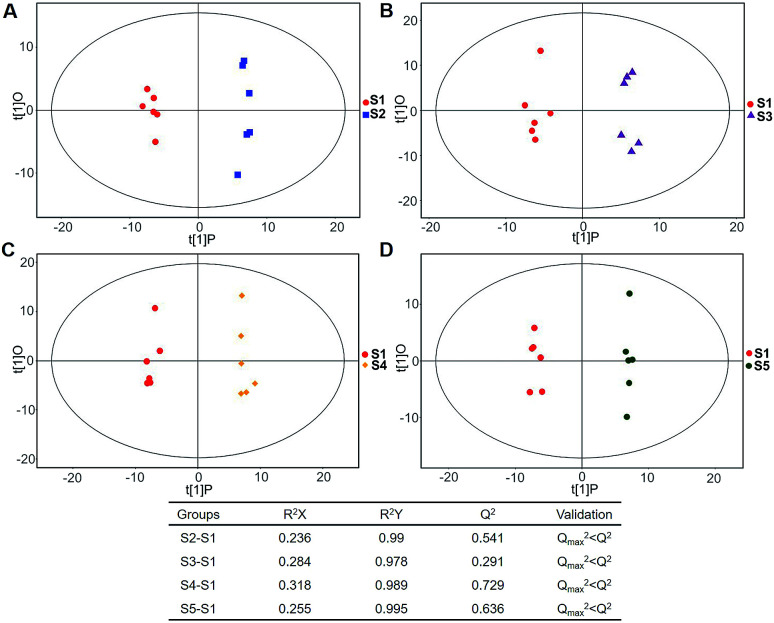
Plots of OPLS-DA and validation of the OPLS-Da model (dynamic analysis of serine). (A) Day 20 KWG (S2) *vs.* day 0 KWG (S1); (B) day 40 KWG (S3) *vs.* day 0 KWG (S1); (C) day 50 KWG (S4) *vs.* day 0 KWG (S1); (D) day 80 KWG (S5) *vs.* day 0 KWG (S1). *R*^2^*X* and *R*^2^*Y* are the cumulative modelled variation in the *X* and *Y* matrix, respectively; *Q*^2^ is the cumulative predicted variation in the *Y* matrix; the validation was same with the description in Fig. 3.

**Fig. 6:**
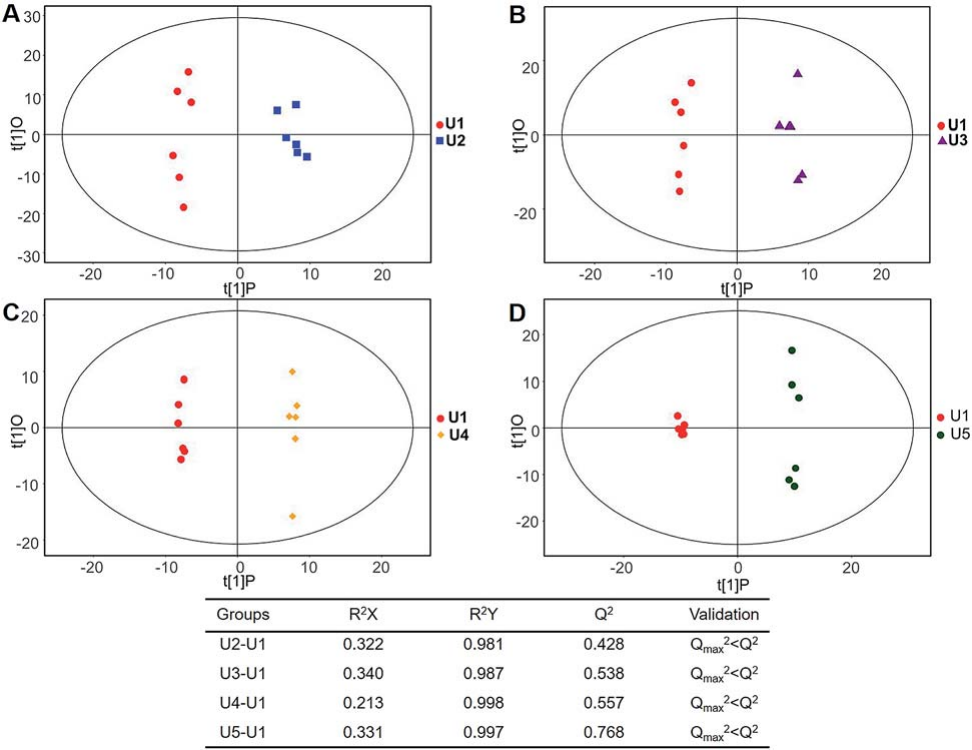
Plots of OPLS-DA and Validation of the OPLS-Da model (dynamic analysis of urine). (A) Day 20 KWG (U2) *vs.* day 0 KWG (U1); (B) day 40 KWG (U3) *vs.* day 0 KWG (U1); (C) day 50 KWG (U4) *vs.* day 0 KWG (U1); (D) day 80 KWG (U5) *vs.* day 0 KWG (U1). *R*^2^*X* and *R*^2^*Y* are the cumulative modelled variation in the *X* and *Y* matrix, respectively; *Q*^2^ is the cumulative predicted variation in the *Y* matrix; the validation was same with the description in Fig. 3.

In total, 167 (serum) and 232 (urine) metabolites were identified by referring to the LECO-Fiehn Rtx5 database (ESI Tables S2 and S3,† respectively). Further statistical analysis was performed on the 26 comparison groups (shown as Fig. 1). Supervised multivariate data analysis (PCA) was used to identify the outliers. However, no obvious separations could be identified in each experimental group comparisons (ESI Fig. 4S–7S†). The application of the OPLS-DA model demonstrated clear separations between each metabolite in the KWG and the CG at each time point (Fig. 3 and 4) and dynamic change comparison groups for KWG (Fig. 5 and 6). Further permutation tests (*n* = 200) were performed to validate the OPLS-DA model. As overfitting was observed in the 20 d and the 40 d time point sample groups of the serological models (*Q*_max_^2^ > *Q*^2^), the two models were considered as lacking reliability and predictability. The fitting of the models for the remaining time point sample groups was considered valid. Thus, further metabolite analysis was performed based on the screening results.

### Metabolites screening and analysis

**Fig. 7:**
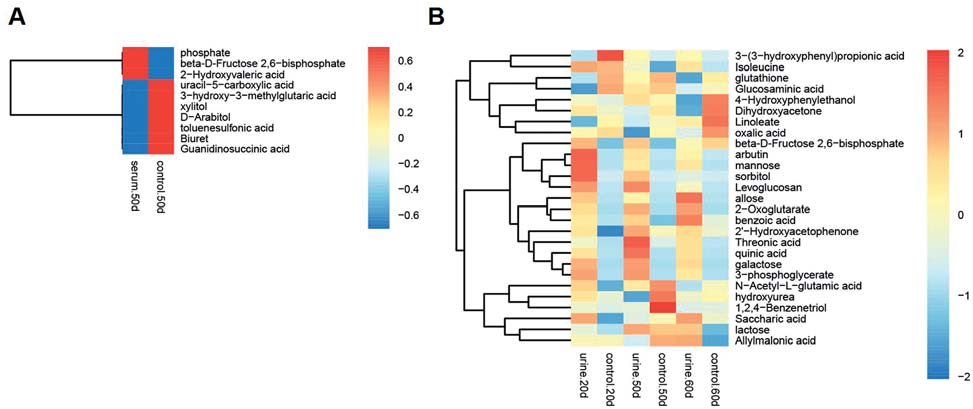
A heat map based on the relative levels of the potential biomarkers in the serum (A) and urine (B) samples in 0–50 days by KW *vs.* CG analysis. The data set was screened using *t*-test *p* value < 0.05, VIP value > 1. Rats in kiwi wine group were treated with kiwi wine with 3.35 mL per kg per day for 50 days, while control group with the same amount water. The data were analysis between KWG and CG.

**Fig. 8:**
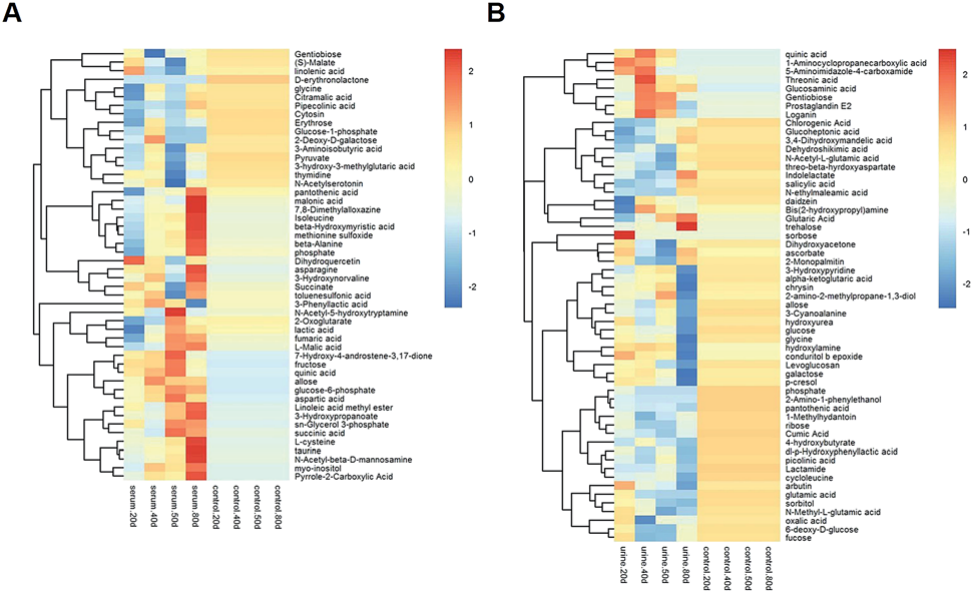
A heat map based on the relative levels of the potential biomarkers in the serum (A) and urine (B) samples in 0–50 days by dynamic analysis. The data set was screened using *t*-test *p* value < 0.05, VIP value > 1. Rats in kiwi wine group were treated with kiwi wine with 3.35 mL per kg per day for 50 days. The data were analysis between every time point and 0 days in KWG.

Multivariate statistical analysis methods were required to analyse the metabolomics data originating the inherent characteristics of GC-TOFMS. Compared with univariate statistical analysis (UVA), such as Student's *t*-test and analysis of variance (ANOVA), multivariate statistical analysis pays more attention to the independent changes in the level of metabolites and the relationship between metabolites and their promotion/antagonism in the biological process. However, we believe cross considering two types of statistical analysis results could help us observe the data from different perspectives and draw conclusions. It could also reduce the false positive errors or overfitting of the model caused by using only one type of statistical analysis method. The selection of metabolites for pathway analysis was based on the principles of the variable importance for the projection (VIP > 1.0)^29^ and the Student's *t*-test (*P* value > 0.05). According to the verification of the models, the changes of serological metabolites became stable after the 40 d time point, while the changes of urinary metabolites could be observed earlier than the 20 d time point. The following metabolite screenings were based on the verification results of the OPLS-DA models. When the metabolism of rats was restored, 10 and 27 metabolites were identified from the screening the serum and urine samples, respectively (Tables S4 and S5†). Sugar metabolism was obviously elevated. 12 serum and 42 urine metabolites were screened to illuminate the metabolic changes after stopping kiwi wine administration (Table S6†). Interestingly, changes in amino acid and fatty acid metabolites were prominent in the KWG. To evaluate the dynamic metabolic changes in rats after 80 days, 52 serum and 57 urine metabolites were screened by comparing each time point samples (20 d, 40 d, 50 d and 80 d) to the control time point (0 d) (Tables S7 and S8,† respectively). In addition, the differences in the metabolites in the control group did not influence the increase in the body weight. Fig. 7 shows a heat map that was generated to visualize the metabolomics changes induced by kiwi wine administration. An overview of the dynamic metabolic changes during the experimental period is provided in Fig. 8.

### Integrated pathway analysis

To better understand the metabolic function of the identified metabolites, the pathways of each metabolite were obtained from the public databases KEGG (http://www.genome.jp/kegg/) and NIST (http://www.nist.gov/index.html). Further comprehensive analyses (including enrichment analysis and topology analysis) by MetaboAnalyst were performed to reveal the highest correlated metabolite pathways (Tables S9 and S10†).^30^ 5 metabolic pathways were enriched in the KWG relative to the CG (*P* < 0.05). As shown in Table 2, 7 metabolites were enriched in 7 metabolic pathways in the KWG in comparison to the CG (*P* < 0.05), and these could be potentially used as biomarkers. Several key metabolic pathways were associated with energy metabolism (fructose and mannose metabolism, galactose metabolism, d-glutamine and d-glutamate metabolism, biosynthesis of phenylpropanoids, tryptophan metabolism, and glycerolipid metabolism) demonstrating that the metabolic changes were attributed to the exogenous wine intake. However, as shown in Table 3, after stopping kiwi wine feeding, the carbohydrate-, amino acid- and fat-related metabolites were still maintained at higher levels. We speculate that the kiwi wine consumption impact on carbohydrate, amino acid, and fat metabolism is long lasting.

**Table 2:** Representative metabolites classified by metabolic pathways in 0–50 days

Metabolic pathway	Serum metabolites	Urine metabolites
**KWG *vs.* control**		
Fructose and mannose metabolism	—	Mannose↑, sorbitol↑, d-fructose 2,6-bisphosphate↑
Galactose metabolism	—	Lactose↑, sorbitol↑, mannose↑
Glycerolipid metabolism	—	3-Phosphoglycerate↑, dihydroxyacetone↓
Linoleic acid metabolism	—	Linoleate↓
d-Glutamine and d-glutamate metabolism	—	2-Oxoglutarate↑

**Dynamic perspective supplement (20, 40, 50 days *vs.* 0 days)**		
Citrate cycle (TCA cycle)	2-Oxoglutarate↓, pyruvate↓, succinate↑, (*S*)-malate↑	—
Butanoate metabolism	2-Oxoglutarate↓, pyruvate↓, succinate↑	—
Alanine, aspartate and glutamate metabolism	2-Oxoglutarate↓, pyruvate↓, succinate↑	—
Glycine, serine and threonine metabolism	Pyruvate↓, tryptophan↓, glycine↓	—
Pyruvate metabolism	Pyruvate↓, succinate↑, (*S*)-malate↑	
Tryptophan metabolism	Tryptophan↓, *N*-acetylserotonin↑	—
d-Glutamine and d-glutamate metabolism	2-Oxoglutarate↓	—

**Table 3:** Representative metabolites classified by metabolic pathways in 80 days

Metabolic pathway	Serum metabolites	Urine metabolites
**KWG *vs.* control**		
Glycine, serine and threonine metabolism	Creatine↓, l-cysteine↑, d-glycerate↑	—

**Dynamic perspective supplement (80 days *vs.* 0 days)**		
Beta-alanine metabolism	Beta-alanine↑, 3-hydroxypropanoate↑	—
Propanoate metabolism	Beta-alanine↑, 3-hydroxypropanoate↑	—
Pantothenate and CoA biosynthesis	Beta-alanine↑, l-cysteine↑	—
Taurine and hypotaurine metabolism	Taurine↑, l-cysteine↑	—
Galactose metabolism	—	Sorbitol↓, glucose↓
d-Glutamine and d-glutamate metabolism	—	2-Oxoglutarate↓

## Discussion

As a popular agricultural product in Asian countries, especially in China and Korea, kiwifruit wine was considered possessing hydroxyl radical scavenging ability and other health promoting functions.^31^ Our pervious analysis (Table S1†) and other researches^32^ have shown that organic acids and sugars are the main constituents of the kiwi wine. However, excessive consumption of sugars has always been linked to several metabolic abnormalities and adverse health conditions.^21^ On the other hand, the active ingredients we detected here or in our previous study are associated with health benefits. For example, polyphenol, ascorbic acid, and flavonoids are claimed to exert beneficial health effects *via* their antioxidant activities.^33,34^ Moreover, small molecular weight amino acids that are produced by the fermentation process of kiwi wine can act as metabolic regulators when used as nutritional supplements.^32^

To better understand the potential health effects of kiwi wine, the present study describes for the first time the metabolomics changes associated with long-term consumption of kiwi wine in rats. After GC-MS/TOF analysis and a cross screening of multivariate analysis and univariate analysis, in the first period of experimentation (0 day to 50 days), identified 7 differentially expressed endogenous serum metabolites and 8 urine metabolites. These metabolites were enriched in several pathways including the citrate cycle (TCA cycle), pyruvate metabolism, fructose and mannose metabolism, galactose metabolism, alanine, aspartate and glutamate metabolism, linoleic acid metabolism and other pathways. These pathways were classified into carbohydrate, amino acid, fat metabolism and other metabolic pathways and were further studied by KEGG analysis. The second experimentation period (50 to 80 days) represented the metabolic regression changes after stopping kiwi wine administration. 6 serum and 3 urine endogenous metabolites were involved in seven pathways including galactose metabolism, beta-alanine metabolism, taurine and hypotaurine metabolism and other pathways. Interestingly, a large part of the metabolites regressed to normal levels after stopping wine supplementation. However, a small part of carbohydrates related to amino acid and fat metabolism were still maintained at higher levels. This result might be associated with the long-term health care effects of kiwi wine.

During first period of experimentation period (kiwi wine supplementation), two aspects of metabolism related information were obtained from the pathway's analysis model (KWG *vs.* CG and dynamic analysis). Notably, the main excrete profile changes were observed in the KWG comparison to the CG; while the dynamic analysis (every time point compared to the 0 day) in the KWG was used to construct the serological metabolic profile changes in rats.

**Fig. 9:**
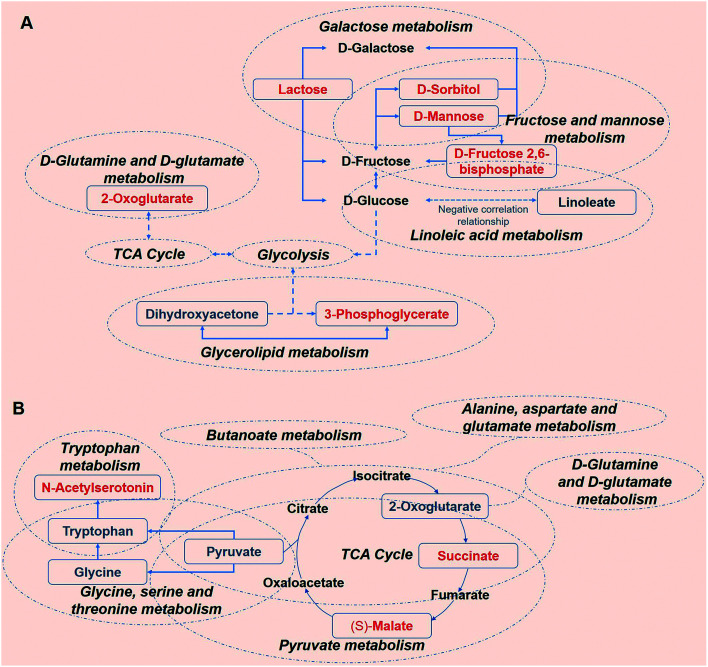
The metabolism network of highest correlation metabolites pathways and relative metabolites in 0–50 days. (A) Urine sample (B) serum. The potential biomarkers that increased are labeled in red and those that decreased are labeled in blue.

Sustained consumption of kiwi wine for 20 days, we primary observed elevated levels of mannose, sorbitol and beta-d-fructose 2,6-bisphosphate (Fru-2,6-P_2_) in the urine samples of the experimental group (Fig. 9A). Fru-2,6-P_2_ was produced as a metabolic by-product generated from ATP and fructose-6-phosphate by 6-phosphofructo-2-kinase and degraded to fructose-6-phosphate and phosphate ion.^35,36^ As high levels of Fru-2,6-P_2_ increase glycolysis and suppress gluconeogenesis, it might be the terminal signal molecule that responds to changes in the nutritional state of the carbohydrate metabolic pathway.^36^ Mannose and sorbitol can be produced from glucose^37,38^ or directly obtained from kiwi wine. The increased urine levels of mannose and sorbitol might be mainly attributed to the extra consumption of kiwi wine. It has been reported that the two constituents do not have adverse effects on health.^39–42^ As shown in the ESI Table S1,† lactose (in galactose metabolism pathway) might have originated from the fermenting process by *Saccharomyces cerevisiae* in the kiwi wine. The up-regulated levels of lactose were mainly due to kiwi wine intake, thus extra lactose was excreted in the urine sample.^43,44^ The changes of above metabolism pathways indicate that consumption kiwi wine might have positive effects on glycolysis.

In the glycerolipid metabolic pathway, 3-phosphoglycerate levels were up-regulated, whereas the levels of dihydroxyacetone were down-regulated. Glucose, pyruvate and alanine contribute to the formation of 3-phosphoglycerate *via* the process of glycolysis and glyceroneogenesis.^45^ Dihydroxyacetone is an intermediate product of the glucose metabolism and plays an important role in regulating the balance between glucose and lipid metabolism.^46^ In the present study, the decline of the urine dihydroxyacetone levels in the KWG is likely due to the phosphorylation of dihydroxyacetone, which generates dihydroxyacetone phosphate and then is converted to 3-phosphoglycerate.^47^ Linoleate that belongs to the linoleic acid metabolic pathway, exhibited reduced urine levels, which indicated a decline in the excretion process after kiwi wine administration (ESI Table S5†). Linoleate has been suggested to have a negative correlation with glucose metabolism.^48^ As described above, the increased levels of 3-phosphoglycerate and decreased levels of linoleate might indicate an up-regulation of carbohydrate metabolism.

d-Glutamine and d-glutamate metabolic pathway was identified because it displayed the lowest *P* value (*P* < 0.05, ESI Table S9†). In this pathway, the identified metabolite was 2-oxoglutarate, which is a key intermediate product of the tricarboxylic acid (TCA) cycle.^49^ 2-Oxoglutarate can be produced by oxidative decarboxylation of isocitrate dehydrogenase, and by deamination of glutamate by glutamate dehydrogenase. Other components of the TCA cycle, such as succinic acid and isocitric acid, together with various carbohydrate substances that are involved in energy metabolism, were detected in the kiwi wine (ESI Table S1†). Thus, consumption of kiwi wine might be a factor for the increased urine levels of 2-oxoglutarate. All together, these results indicate that kiwi wine primary affects the energy metabolism pathway, especially the carbohydrate pathway.

From the dynamic perspective, during the 50 days period, the effects of kiwi wine consumption to the experimental group were mainly reflected on the TCA cycle related pathways. Based on their *p* value and impact factor (ESI Table S9†), the screened pathways can be ordered as follows: TCA cycle, butanoate metabolism, alanine, aspartate and glutamate metabolism, glycine, serine and threonine metabolism, tryptophan metabolism, d-glutamine and d-glutamate metabolism and pyruvate metabolism. The representative metabolites of these pathways that identified in our study are 2-oxoglutarate, succinate, (*S*)-malate, pyruvate, tryptophan, *N*-acetylserotonin, and glycine (Fig. 9B).

Among these metabolites, 2-oxoglutarate, pyruvate, tryptophan and glycine were down-regulated in the serum. Notably, the excreted levels of 2-oxoglutarate in urine were up-regulated in the KWG when compared to CG, while in the serum an opposite result was observed. TCA cycle is not only the final metabolic pathway of three major nutrients (carbohydrates, lipids, and amino acids), but also the link between sugar, lipid, and amino acid metabolism, and the main way to obtain energy for the body.^50^ The observed changes in the levels of 2-oxoglutarate indicate that kiwi wine can affect the energy metabolism. Succinate and (*S*)-malate are all the metabolic intermediates of the TCA cycle and its related pathways. The up-regulation of these metabolites suggests that the long-term consumption of kiwi wine mainly promotes the carbohydrate metabolism in rats.

Pyruvate is a key intermediate product of several metabolic pathways, which can be converted into carbohydrates *via* gluconeogenesis, to fatty acids or energy through acetyl-CoA, and to amino acids, and ethanol.^51^ As seen in Fig. 9B, pyruvate links several metabolite pathways, and connects glycolysis with other processes such as alanine, aspartate, glutamate, glycine, serine, and threonine metabolism. The reduced expression of pyruvate in the serum denotes glycolysis activation and higher energy conversion requirement in rats. The small dynamic reduction of tryptophan in the serum might be related to the synthesis of serotonin,^52^ which is further supported by the increased serum expression levels of another significant metabolite as *N*-acetylserotonin. *N*-acetylserotonin is a naturally occurring chemical precursor and intermediate in the endogenous production of melatonin from serotonin, and is considered as a potent antioxidant agent.^53,54^ The declined levels of glycine were only observed on the 20th day, which might have been caused by a primary nutrition disturbance from kiwi wine.

Collectively, the results from both statistical analysis models revealed that consumption of kiwi wine can primary effect carbohydrate metabolism and its related metabolites. This effect is mainly attributed to the sugar contents in the kiwi wine. Nonetheless, the sugar content of kiwi wine can potentially promote the energy metabolism and can have other beneficial effects on health.

**Fig. 10:**
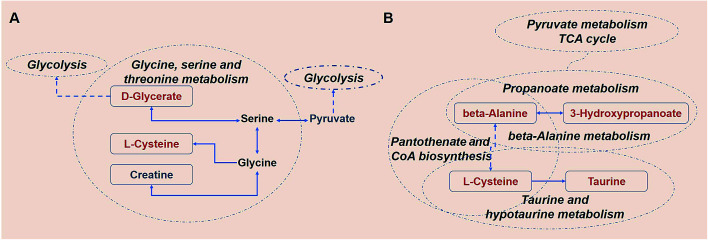
The metabolism network of highest correlation metabolites pathways and relative metabolites in 80 days. (A) KWG *vs.* CG (B) dynamic analysis. The potential biomarkers that increased are labeled in red and those that decreased are labeled in blue.

After 80 days, some metabolic changes in amino acids and lipids could be still observed (Fig. 10). The levels of the serological metabolites creatine, l-cysteine, and d-glycerate were altered in the KWG relative to the CG. These three metabolites are all intermediate products of the glycine, serine and threonine metabolic pathway. Creatine is naturally produced from the amino acid glycine.^55^ Thus, the minor down-regulation of creatine might be caused by the cease of kiwi wine supplementation (ESI Table S1†). As a precursor of several important biochemical intermediates in glycolysis, the increased expression of d-glycerate indicates a sustained impact on glycolysis related pathways by kiwi wine (Fig. 10A). Thus, the basal metabolism of rats was likely improved.

Further information was obtained from the dynamic analysis (Fig. 10B). The beta-alanine metabolism and propanoate metabolism were still sustained at higher levels. The propanoate metabolic process is involved in crucial *in vivo* metabolic pathways and is associated with the TCA cycle.^56^ In addition, beta-alanine is a precursor of the oxidative substrate acetyl-coenzyme A.^57^ Pathway screening analysis showed that pantothenate and acetyl-CoA biosynthesis was another key metabolic pathway implicated in the response to kiwi wine consumption, with the prominent serological metabolites being beta-alanine and l-cysteine. Up-regulation of l-cysteine was found in both the KWG and the CG and dynamic level changes were also observed (ESI Tables S6 and S7†). Recently there is a growing interest in the use of l-cysteine for improving the health of animals and humans, due to its beneficial effects on oxidative stress, gut function, lipid metabolism, and animal growth performance (food intake, body weight gain, and feed efficiency).^58^ In addition, serum taurine and l-cysteine levels incremented after 80 days, both of which are related to the taurine and hypotaurine metabolic pathway. Taurine is an end product of l-cysteine metabolism.^59^ Moreover, taurine has been shown to act as a protective agent against several environmental toxins and to prevent drug-induced organ dysfunction and diabetes. Thus, an increased conversion of l-cysteine to taurine might provide a novel insight for the nutritional value of l-cysteine and its therapeutic potential.^60^ As a conclusion, sustained consumption of kiwi wine might activate the l-cysteine related pathway and promote some nutritional benefits.

80 days later, the carbohydrates were converted to other metabolites (amino acids and lipids) in the KWG. The decline observed in the urine levels of 2-oxoglutarate, sorbitol and glucose (ESI Table S8†), might indicate their increased conversion to other metabolites. The level of metabolites excreted reflects both the adequacy of the diet and the degree of body health, especially for 2-oxoglutarate,^61^ although there is no direct evidence to link the declined excretion of these metabolites with health. According to previous studies, the increased excretion of 2-oxoglutarate, sorbitol and glucose are related to metabolism-associated diseases like diabetes,^61,62^ interstitial cystitis/painful bladder syndrome,^63^ gastric cancer,^64^ and polycystic kidney disease.^65^ Moreover, Ekblond *et al.*^66^ have proposed that the declined levels of urinary glucose excretion might serve as an evaluation factor for health protection. As mentioned above, the health effect of kiwi wine could be sustained for a long period of time after consumption has ceased. The potential health promoting effects of kiwi wine are mainly reflected on the l-cysteine-related nutritional benefits, which can maintain higher levels of glycolysis and provide health protection *via* reducing the levels of glucose excretion.

Further deeper data mining analysis revealed that kiwi wine intake might influence the microbiota and the antioxidant metabolism. As a representative, serum dihydroquercetin (ESI Table S7†), along with urine glutathione (ESI Table S5†), chlorogenic acid, and loganin (ESI Table S8†) were screened *via* OPLS-DA models analysis. Glutathione is synthesized from the amino acids l-cysteine, l-glutamic acid and glycine and in rats it is associated with the gut microbiota.^67^ Dihydroquercetin, chlorogenic acid, and loganin, are present in vegetarian foods.^68–70^ Interestingly, the absorption and metabolism of several antioxidant constituents (including chlorogenic acid and loganin) are depended on the gut microbiota.^69,71,72^ However, the above metabolites cannot be considered as biomarkers of altered flora metabolism caused by kiwi wine. The direct biomarkers of altered microbiota metabolism were not detected in this study, under the restricted detection range used for GC-MS/TOF analysis. Furthermore, intake of kiwi wine might induce a minor increase in body weight. The weight gain of KWG rats maintained a steady increased trend, while the weight of CG rats fluctuated during the experimental periods. It can be speculated that in KWG rats, kiwi wine promoted stronger adaptability to the changes in the surrounding environment.

Although a popular kiwi fermentation product, the potential health benefits of kiwi wine have rarely been researched. In this study, the health effects of kiwi wine were studied based on a metabonomics-based approach and mathematical statistics models' analysis in rats. To the best of our knowledge, this is the first comprehensive metabolomics approach to study the health effects of kiwi wine. However, a limitation of our study is that the GC-MS/TOF detection range might restrict the identification of more metabolites with altered expression. Other study limitations are the small sample size, and since the main metabolic constituent was sugar, changes in insulin levels should have also been analysed. Therefore, more comprehensive metabolomics studies with larger sample size by liquid chromatography-mass spectrometry (LC-MS) or similar techniques are required. In addition, the dose and frequency of wine-consumption is a controversial topic. In this research, the WSET guideline was utilized as a reference for primary study of the health effect of kiwi wine. Although it seems that the negative effects of alcohol were not shown in this study. Precise quantitative experiments and the adverse effect of alcohol should be considered in future.

## Conclusions

In conclusion, our present study showed that the consumption of kiwi wine could be partly possess health promoting function *via* a long-term metabolism effect. Moderate kiwi wine consumption can alter the energy metabolism (carbohydrate metabolism), and cause a sustained effect on energy, amino acid and lipid metabolism. These results provide a nutrition field view for the development of kiwi wine agricultural industry.

## Conflicts of interest

There are no conflicts to declare.

## Supplementary Material

RA-009-C9RA02138H-s001
