# Supplementary material for: Health effects of kiwi wine on rats: an untargeted metabolic fingerprint study based on GC-MS/TOF
Source: RSC Adv. 2019 May 3;9(24):13797–807. doi: 10.1039/c9ra02138h (PMC9063974; doi:10.1039/c9ra02138h)
Supplement: RA-009-C9RA02138H-s001 [file RA-009-C9RA02138H-s001.pdf]

Electronic Supplementary Material (ESI) for RSC Advances.
This journal is © The Royal Society of Chemistry 2019

Supporting information

Table of contents

-Details of wine sample preparation and detection

-Quality control evaluation of samples

- Supplementary tables

- Supplementary figures

Wine sample preparation and detection:

Sample preparation for GC-MS analysis.

Take 100 μL kiwi wine sample into 1.5 mL EP tubes. Add 0.35 mL methanol to the sample. Vortex mixing for 10 seconds; Centrifuge for 15 min at 13000 r/min, 4 °C; Transfer the supernatant (0.39 mL) into a fresh 2 mL GC/MS glass vial. Derivatization reactions were taken to modify the wine samples. Take wine samples and dry in a vacuum concentrator without heating; Add 80 μL of methoxy amination hydrochloride (20 mg/mL in pyridine), incubated for 30 min at 80°C; Add 100 μL of the BSTFA regent (1 % TMCS, v/v) to the sample aliquots, incubated for 1.5 hours at 70 °C; Mix well for GC-MS analysis.

GC-MS analysis.

And for kiwi wine samples, 2 μL aliquot of the analyte was injected in splitless mode. Helium was used as the carrier gas, the front inlet purge flow was 3 mL/min, and the gas flow rate through the column was 1 mL/min. The initial temperature was kept at 50 °C for 1 min, then raised to 310 °C at a rate of 10 °C/min, then kept for 3 min at 310 °C. The injection, transfer line, and ion source temperatures were 280, 270, and 220 °C, respectively. The energy was -70 eV in electron impact mode. The mass spectrometry data were acquired in full-scan mode with the m/z range of 50-500 at a rate of 20 spectra per second after a solvent delay of 455 seconds.

Constituents of kiwi wine by GC-MS/TOF.

Supporting Table S1 Constituents of kiwi wine

No.	R.T.	Name	Percent %	Molecular formula
1	9.0175	Lactic acid	0.5603	$C_3H_5O_2$
2	9.26583	Glycolic acid	0.0286	$C_2H_4O_3$
3	9.63583	Alanine	0.037	$C_3H_7NO_2$
4	10.05	Glycine	0.0014	$C_2H_5NO_2$
5	10.14	Oxalic acid	0.0202	$H_2C_2O_4$
6	10.2925	3-Hydroxypropionic acid	0.1369	$C_3H_6O_3$
7	10.63	Lactamide	0.2755	$C_3H_7NO_2$
8	11.9367	Serine	0.0013	$C_3H_7NO_3$
9	12.4067	2-Deoxy erythritol	0.3804	$C_4H_{10}O_3$
10	12.4625	L-Allothreonine	0.0059	$C_4H_9NO_3$
11	12.5892	Maleic acid	0.0412	$C_4H_4O_4$
12	12.5975	Threonine	0.0012	$C_4H_9NO_3$
13	12.7225	Succinic acid	0.1184	$C_4H_6O_4$
14	12.8925	Catechol	0.1345	$C_6H_6O_2$
15	12.9283	Thymol	0.0115	$C_{10}H_{14}O$
16	13.1458	Allylmalonic acid	0.0987	$C_6H_6O_4$
17	13.2783	Itaconic acid	0.0398	$C_5H_6O_4$
18	13.7292	Tartronic acid	0.0027	$C_3H_4O_5$
19	13.87	4-Methylcatechol	0.0058	$C_7H_8O_2$
20	14.3667	Erythrose	0.0080	$C_7H_{10}O_4$
21	14.5075	D-Erythronolactone	0.0048	$C_4H_6O_4$
22	14.5192	L-Threose	0.0152	$C_4H_8O_4$
23	15.1392	Threitol	0.2115	$C_4H_{10}O_4$
24	15.3225	1,5-Anhydroglucitol	0.0349	$C_6H_{12}O_5$
25	15.3975	Adipic acid	0.6691	$C_6H_{10}O_4$
26	15.4083	3-Hexenedioic acid	0.0085	$C_6H_8O_4$
27	15.4558	Asparagine	0.0079	$C_4H_8N_2O_3$
28	15.6217	Acetol	0.1343	$C_3H_6O_2$
29	15.6542	Methionine	0.0082	$C_5H_{11}O_2NS$
30	15.7383	L-Glutamic acid	0.003	$C_5H_9NO_4$
31	15.7433	Pyrogallol	0.3437	$C_6H_6O_3$
32	15.81	Cumic Acid	0.0126	$C_{10}H_{12}O_2$
33	15.9125	Creatine	0.0233	$C_4H_9N_3O_2$
34	16.01	(2R,3S)-2-Hydroxy-3-isopropylbutanedioic acid	0.0120	$C_7H_{12}O_5$
41	16.0742	2-Hydroxy-2-phenylacetic acid	0.0097	$C_8H_8O_3$
42	16.1	N-Methylanthranilic acid	0.0149	$C_8H_9NO_2$
43	16.275	3-Phenyllactic acid	0.075	$C_9H_{10}O_3$

44	16.2825	threo-beta-Hyrdoxy aspartate	0.0023	$C_4H_7NO_5$
45	16.4033	3-Hydroxy-3-methylglutaric acid	0.0045	$C_6H_{10}O_5$
46	16.4608	1,2,4-Benzenetriol	0.1079	$C_6H_6O_3$
47	16.755	Tartaric acid	0.0021	$C_4H_6O_6$
48	16.9167	Lyxose	0.1463	$C_5H_{10}O_5$
49	16.9342	Phloroglucinol	0.0129	$C_6H_6O_3$
50	16.9792	Allose	0.1719	$C_6H_{12}O_6$
51	17.0142	Xylose	0.0289	$C_5H_{10}O_5$
52	17.0225	4-Hydroxyphenylacetic acid	0.003	$C_8H_8O_3$
53	17.115	Creatine degr	0.0343	-
54	17.2592	Ribose	0.0058	$C_5H_{10}O_5$
55	17.345	N-Methyl-L-glutamic acid	0.0047	$C_6H_{12}ClNO_4$
57	17.3892	Ribonic acid gamma-lactone	0.0111	$C_5H_8O_5$
58	17.5142	3,6-Anhydro-D-galactose	0.2104	$C_6H_{10}O_5$
59	17.6258	Levoglucosan	0.1092	$C_6H_{10}O_5$
60	17.795	D-Arabitol	0.0452	$C_5H_{12}O_5$
62	17.8058	Ribitol	0.3064	$C_5H_{12}O_5$
63	17.9217	Dihydrocoumarin	0.0013	$C_9H_8O_2$
64	17.9258	Fucose	0.0037	$C_6H_{12}O_5$
65	18.0017	Lyxonic acid-1,4-lactone	0.0548	$C_5H_8O_5$
67	18.1367	Diglycerol	0.1441	$C_3H_8O_3$
68	18.1875	Glucose-1-phosphate	0.1023	$C_6H_{13}O_9P$
69	18.2733	5,6-Dimethylbenzimidazole	0.203	$C_9H_{10}N_2$
70	18.4808	2-Deoxy-D-galactose	0.4745	$C_6H_{12}O_5$
71	18.5942	Shikimic acid	0.564	$C_7H_{10}O_5$
72	18.8325	trans-2-Hydroxycinnamic acid	0.0085	$C_9H_8O_3$
73	18.8817	alpha-D-Glucosamine 1-phosphate	0.0043	$C_6H_{14}NO_8P$
74	18.9575	Isocitric acid	0.0027	$C_6H_8O_7$
75	19.0325	Tagatose	1.2991	$C_6H_{12}O_6$
76	19.0533	Methyl-beta-D-galactopyranoside	0.7597	$C_7H_{14}O_6$
77	19.1058	1,5-Anhydroglucitol	0.2245	$C_6H_{12}O_5$
78	19.1508	Quinic acid	0.1795	$C_7H_{12}O_6$
79	19.2033	beta-Mannosyl glycerate	1.6147	$C_9H_{18}O_9$
80	19.4058	Sorbose	0.0567	$C_6H_{12}O_6$
81	19.4658	Fructose	0.0644	$C_6H_{12}O_6$
82	19.4917	D-Talose	5.1032	$C_6H_{12}O_6$
83	19.7133	Galactose	0.2574	$C_6H_{12}O_6$
84	19.7267	Mannose	0.2171	$C_6H_{12}O_6$
85	19.7858	Gluconic lactone	0.5828	$C_6Hl_0O_6$
86	19.8433	L-Gulonolactone	0.6606	$C_6H_{10}O_6$
87	19.8708	Gluconic acid	1.3412	$C_6H_{12}O_7$
88	19.9992	Sorbitol	0.1402	$C_6H_{14}O_6$

89	20.0033	Sedoheptulose	0.0887	$C_7H_{12}O_6$
90	20.0325	Mannitol	0.0011	$C_6H_{14}O_6$
91	20.1067	Conduritol b epoxide	0.0354	$C_6H_{10}O_5$
92	20.2667	Gallic acid	0.0205	$C_7H_6O_5$
93	20.5558	3,5-Dihydroxyphenylglycine	0.0006	$C_8H_9NO_4$
94	20.63	Galactonic acid	0.0049	$C_6H_{10}O_7$
95	20.6808	N-alpha-Acetyl-L-ornithine	0.0013	$C_7H_{14}N_2O_3$
96	20.7642	Saccharic acid	0.0034	$C_6H_{12}O_7$
97	20.8892	beta-Hydroxymyristic acid	0.0025	$C_{14}H_{28}O_3$
98	21.4458	myo-Inositol	0.4755	$C_6H_{12}O_6$
99	21.8308	Glucoheptonic acid	0.0218	$C_7H_{14}O_8$
100	22.0717	d-Glucoheptose	0.0304	$C_7H_{14}O_7$
101	24.2425	Resveratrol	0.0028	$C_{14}H_{12}O_3$
102	24.7592	cytidine-Monophosphate degr prod	0.007	-
103	25.0617	Salicin	0.0133	$C_{13}H_{18}O_7$
104	25.3058	Piceatannol	0.0025	$C_{14}H_{12}O_4$
105	25.4467	Arbutin	0.0089	$C_{12}H_{16}O_7$
106	25.8817	Sucrose	0.2112	$C_{12}H_{22}O_{11}$
107	26.3067	Xanthosine	0.001	$C_{10}H_{12}N_4O_6$
108	26.4558	Lactulose	0.0205	$C_{12}H_{22}O_{11}$
109	26.4625	Lactose	0.0088	$C_{12}H_{22}O_{11}$
110	26.5592	Cellobiose	0.0095	$C_{12}H_{22}O_{11}$
111	26.705	Maltose	0.0053	$C_{12}H_{22}O_{11}$
112	26.7925	Trehalose	0.2354	$C_{12}H_{22}O_{11}$
113	26.9	Lactobionic Acid	0.0469	$C_{12}H_{22}O_{12}$
114	26.9725	Cellobiotol	0.0059	$C_{12}H_{24}O_{11}$
115	27.23	Gentiobiose	0.0203	$C_{12}H_{22}O_{11}$
116	27.6783	Melibiose	0.0063	$C_{12}H_{24}O_{12}$
117	27.7633	Digalacturonic acid	0.0121	$C_{12}H_{18}O_{13}$

Quality control evaluation of samples

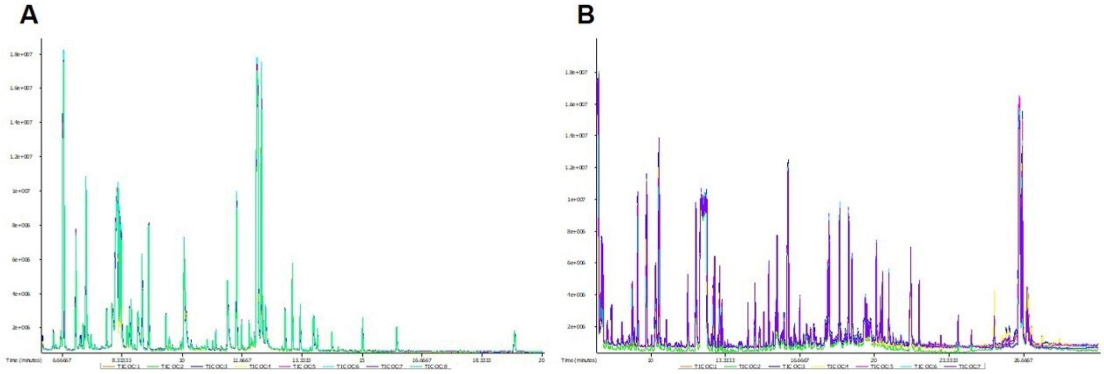

Supporting Figure. S1 Typical ion chromatograms (TIC) of the serum sample (A) and urine samples (B)

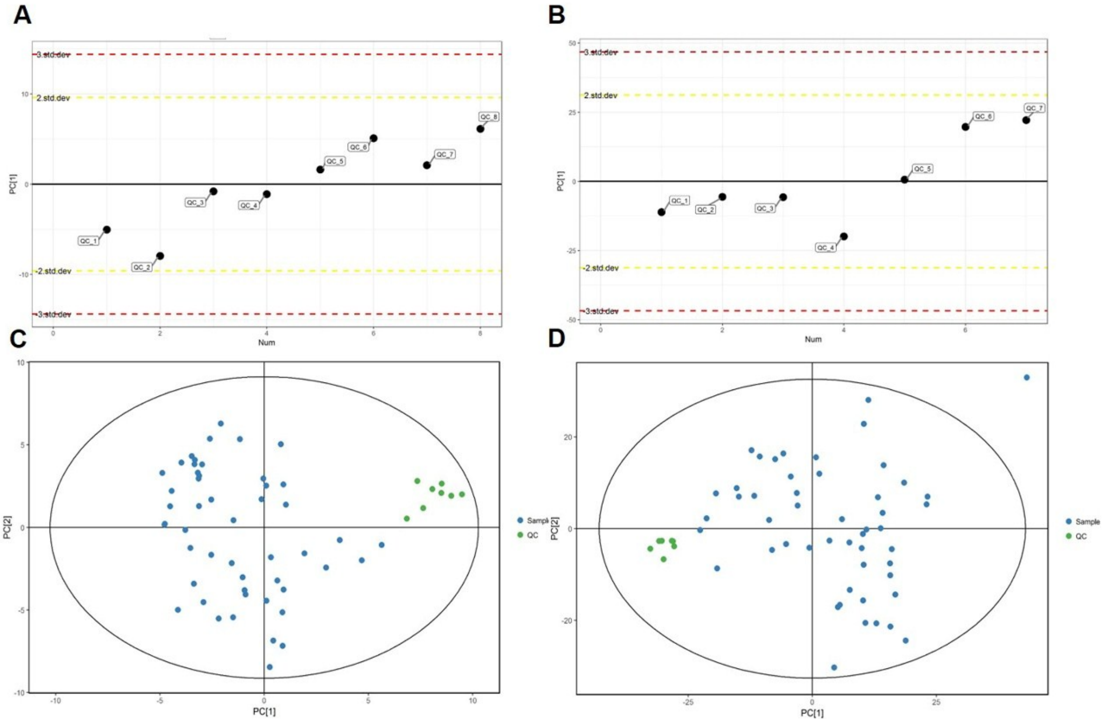

Supporting Figure. S2 Principal Component Analysis of QC samples

Serum sample 1D(A) ; Urine samples 1D(B); Serum sample 2D(C) ; Urine samples 2D(D);

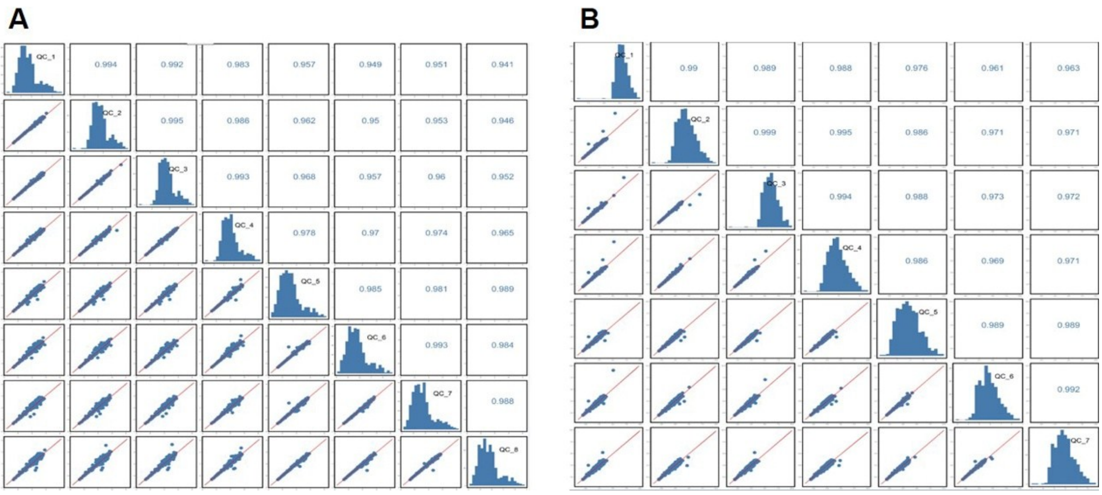

Supporting Figure. S3 The correlation of QC samples

Serum sample (A); Urine samples (B)

The typical ion chromatograms chart showed that the well overlapped route time and peak area indicated the stability of the instrument. The peak area of internal standard (RSD < 30 %) exhibited the stability of instrument data acquisition. There was no cross contamination between samples via examining the blank sample. The stability of analysis method was provided by the 1D and 2D principal component analysis of QC samples and the correlation of QC (closed to 1). As a conclusion, the quality of serum and urine samples, the analysis method and the stability of system were suitable for the experiment.

Supporting Table S2 Metabolites in serum detected by GC-MS/TOF

No	Name	R.T.	MW	Molecular formula
1	2-Hydroxypyridine	6.6221	95	C_5H_5NO
2	Pyruvic acid	6.6365	88	$C_3H_4O_3$
3	Lactic acid	6.7071	90	$C_3H_6O_3$
4	Glycolic acid	6.8277	76	$C_2H_4O_3$
5	Alanine	7.0389	89	$C_3H_7NO_2$
6	Hydroxylamine	7.1915	33	H_3NO
7	Oxalic acid	7.2087	90	$C_2H_2O_4$
8	3-Hydroxybutyric acid	7.4679	104	$C_4H_8O_3$
9	N-Methyl-DL-alanine	7.5735	103	$C_4H_9NO_2$
10	Benzyl alcohol	7.6072	108	C_7H_8O
11	alpha-Ketoisocaproic acid	7.6295	130	$C_6H_{10}O_3$
12	2-Amino-3-methyl-1-butanol	7.6361	103	$C_5H_{13}NO$
13	Methyl Phosphate	7.6630	112	CH_5O_4P
14	Malonic acid	7.7743	104	$C_3H_4O_4$
15	2-Hydroxyvaleric acid	7.8529	118	$C_5H_{10}O_3$
16	Valine	7.8974	117	$C_5H_{11}NO_2$
17	oxamic acid	8.1990	89	$C_2H_3NO_3$
18	Ethanolamine	8.2900	61	C_2H_7NO
19	Phosphate	8.3016	95	O_4P^{-3}
20	Thymidine	8.3500	242	$C_{10}H_{14}N_2O_5$
21	Isoleucine	8.4595	131	$C_6H_{13}NO_2$
22	4-Aminobutyric acid	8.4914	103	$C_4H_9NO_2$
23	Proline	8.5382	115	$C_5H_9NO_2$
24	Glycine	8.5798	75	$C_2H_5NO_2$
25	Succinate	8.6087	118	$C_4H_6O_4$
26	2,3-Dihydroxypyridine	8.6604	111	$C_5H_5NO_2$
27	D-Glycerate	8.6702	106	$C_3H_6O_4$
28	Catechol	8.6993	110	$C_6H_6O_2$
29	Thymol	8.7244	150	$C_{10}H_{14}O$
30	Uracil	8.8203	112	$C_4H_4N_2O_2$
31	Itaconic acid	8.8307	130	$C_5H_6O_4$
32	Fumaric acid	8.8605	116	$C_4H_4O_4$
33	Serine	8.9127	105	$C_3H_7NO_3$
34	Citraconic acid	8.9205	130	$C_5H_6O_4$
35	Pyrrole-2-carboxylic acid	8.9402	111	$C_5H_5NO_2$
36	Pelargonic acid	8.9646	158	$C_9H_{18}O_2$
37	Alanine	8.9691	89	$C_3H_7NO_2$
38	Pipecolinic acid	9.0229	129	$C_6H_{11}NO_2$
39	2,3-Dimethylsuccinic acid	9.0280	146	$C_6H_{10}O_4$

40	3-Hydroxynorvaline	9.0679	133	$C_5H_{11}NO_3$
41	Resorcinol	9.1363	110	$C_6H_6O_2$
42	Thymine	9.2539	126	$C_5H_6N_2O_2$
43	beta-Alanine	9.4024	89	$C_3H_7NO_2$
44	Erythrose	9.4786	158	$C_7H_{10}O_4$
45	L-Homoserine	9.4993	119	$C_4H_9NO_3$
46	2,4-Diaminobutyric acid	9.6236	118	$C_4H_{10}N_2O_2$
47	3-Aminoisobutyric acid	9.6353	103	$C_4H_9NO_2$
48	Aminomalonic acid	9.6479	119	$C_3H_5NO_4$
49	(S)-Malate	9.7268	134	$C_4H_6O_5$
50	Citramalic acid	9.7398	148	$C_5H_8O_5$
51	Threitol	9.8226	124	$C_4H_{10}O_4$
52	3-Hexenedionic acid	9.9191	144	$C_6H_8\ O_4$
53	Aspartic acid	9.9442	133	$C_4H_7NO_4$
54	trans-4-Hydroxy-L-proline	10.0026	131	$C_5H_9NO_3$
55	Methionine	10.0082	149	$C_5H_{11}NO_2S$
56	Oxoproline	10.0564	129	$C_5H_7NO_3$
57	Glutamine	10.1662	146	$C_5H_{10}N_2O_3$
58	L-Cysteine	10.1999	121	$C_3H_7NO_2S$
59	Creatine	10.2464	131	$C_4H_9N_3O_2$
60	3-Hydroxypropanoate	10.2700	90	$C_3H_6O_3$
61	2-Oxoglutarate	10.2971	146	$C_5H_6O_5$
62	(2R,3S)-2-Hydroxy-3-isopropylbutanedioic acid	10.3338	176	$C_7H_{12}O_5$
63	3-Phenyllactic acid	10.4304	166	$C_9H_{10}O_3$
64	3-Hydroxy-3-methylglutaric acid	10.4500	162	$C_6H_{10}O_5$
65	D-Erythronolactone	10.4997	118	$C_4H_6O_4$
66	beta-Glutamic acid	10.5416	147	$C_5H_9NO_4$
67	Glutamic acid	10.5487	147	$C_5H_9NO_4$
68	Toluenesulfonic acid	10.6794	172	$C_7H_8O_3S$
69	Phenylalanine	10.6946	165	$C_9H_{11}NO_2$
70	Xylose	10.7165	152	$C_5H_{12}O_5$
71	Lyxose	10.7193	150	$C_5H_{10}O_5$
72	Cytosin	10.7312	111	$C_4H_5N_3O$
73	Lauric acid	10.8274	200	$C_{12}H_{24}O_2$
74	Allose	10.8388	180	$C_6H_{12}O_6$
75	Asparagine	10.8810	132	$C_4H_8N_2O_3$
76	Taurine	10.9317	125	$C_2H_7NO_3S$
77	Xylitol	11.0910	152	$C_5H_{12}O_5$
78	Guanidinosuccinic acid	11.0311	175	$C_5H_9N_3O_4$
79	Acetol	11.0419	74	$C_3H_6O_2$
80	beta-Glycerophosphoric	11.1198	172	$C_3H_9O_6P$

	acid			
81	D-Arabitol	11.1201	152	$C_5H_{12}O_5$
82	Fucose	11.1603	164	$C_6H_{12}O_5$
83	6-Deoxy-D-glucose	11.2114	164	$C_6H_{12}O_5$
84	Aconitic Acid	11.2677	174	$C_6H_6O_6$
85	3,6-Anhydro-D-galactose 3	11.3086	162	$C_6H_{10}O_5$
86	Putrescine	11.3161	88	$C_4H_{12}N_2$
87	D-Glycerol 1-phosphate	11.3271	172	$C_3H_9O_6P$
88	Glucose-1-phosphate	11.3696	276	$C_6H_{13}O_{10}P$
89	Biuret	11.4195	103	$C_2H_5N_3O_2$
90	Uracil-5-carboxylic acid	11.4429	156	$C_5H_4N_2O_4$
91	Gentisic acid	11.4807	154	$C_7H_6O_4$
92	Methionine sulfoxide	11.5060	181	$C_5H_{11}NO_4S$
93	2-Deoxy-D-galactose	11.5702	164	$C_6H_{12}O_5$
94	2-Deoxy-D-glucose	11.5775	164	$C_6H_{12}O_5$
95	Citric acid	11.6470	192	$C_6H_8O_7$
96	Ornithine	11.6876	132	$C_5C_5H_{12}N_2O_2$
97	Citrulline	11.7156	175	$C_6H_{13}N_3O_3$
98	alpha-D-Glucosamine 1-phosphate	11.7379	259	$C_6H_{14}NO_8P$
99	Isocitric acid	11.7418	192	$C_6H_8O_7$
100	Tagatose	11.7927	180	$C_6H_{12}O_6$
101	beta-Mannosyl glycerate	11.8570	270	$C_9H_{18}O_9$
102	Quinic acid	11.8627	192	$C_7H_{12}O_6$
103	Fructose	11.9150	180	$C_6H_{12}O_6$
104	2,6-Diaminopimelic acid	11.9330	190	$C_7H_{14}N_2O_4$
105	1,5-Anhydro glucitol	11.9496	164	$C_6H_{12}O_5$
106	Mannose	12.0030	180	$C_6H_{12}O_6$
107	Mannose	12.1953	180	$C_6H_{12}O_6$
108	Lysine	12.2371	146	$C_6H_{14}N_2O_2$
109	Glucose	12.2462	180	$C_6H_{12}O_6$
110	Sorbitol	12.2748	182	$C_6H_{14}O_6$
111	D-Talose	12.2790	180	$C_6H_{12}O_6$
112	Tyrosine	12.3380	181	$C_9H_{11}NO_3$
113	Sedoheptulose	12.3669	210	$C_7H_{14}O_7$
114	Conduritol b epoxide	12.3883	162	$C_6H_{10}O_5$
115	Indole-3-acetic acid	12.5619	175	$C_{10}H_9NO_2$
116	Pantothenic acid	12.5837	219	$C_9H_{17}NO_5$
117	Gluconic acid	12.628,0	196	$C_6H_{12}O_7$
118	Galactonic acid	12.6412	196	$C_6H_{12}O_7$
119	beta-Hydroxymyristic acid	12.7362	244	$C_{14}H_{28}O_3$
120	Palmitoleic acid	12.7808	254	$C_{16}H_{30}O_2$
121	Palmitic acid	12.8631	256	$C_{16}H_{32}O_2$

122	N-Acetyl-beta-D-mannosamine	12.9763	221	$C_8H_{15}NO_6$
123	myo-Inositol	13.0599	180	$C_6H_{12}O_6$
124	Xanthotoxin	13.1027	216	$C_{12}H_8O_4$
125	Ferulic acid	13.1350	194	$C_{10}H_{10}O_4$
126	N-Acetyl-beta-D-mannosamine	13.1442	221	$C_8H_{15}NO_6$
127	Linoleic acid methyl ester	13.1818	294	$C_{19}H_{34}O_2$
128	Glucoheptonic acid	13.2483	226	$C_7H_{14}O_8$
129	Heptadecanoic acid	13.3223	270	$C_{17}H_{34}O_2$
130	d-Glucoheptose	13.4116	210	$C_7H_{14}O_7$
131	Indolelactate	13.5009	205	$C_{11}H_{11}NO_3$
132	beta-D-Fructose 2,6-bisphosphate	13.5749	340	$C_6H_{14}O_{12}P_2$
133	Phytanic acid	13.5925	312	$C_{20}H_{40}O_2$
134	Linoleate	13.6395	280	$C_{18}H_{32}O_2$
135	Oleic acid	13.6570	282	$C_{18}H_{34}O_2$
136	Linolenic acid	13.6762	278	$C_{18}H_{30}O_2$
137	Tryptophan	13.6964	204	$C_{11}H_{12}N_2O_2$
138	Stearic acid	13.7598	284	$C_{18}H_{36}O_2$
139	Fructose-6-phosphate	13.9443	260	$C_6H_{13}O_9P$
140	Glucose-6-phosphate	14.0119	260	$C_6H_{13}O_9P$
141	Phenyl beta-D-glucopyranoside	14.0568	256	$C_{12}H_{16}O_6$
142	Arachidonic acid	14.3392	304	$C_{20}H_{32}O_2$
143	6-Phosphogluconic acid	14.4599	276	$C_6H_{13}O_{10}P$
144	cis-Gondoic acid	14.5401	76	$C_2H_4O_3$
145	N-Acetylserotonin	14.5631	218	$C_{12}H_{14}N_2O_2$
146	Arachidic acid	14.6052	304	$C_{20}H_{32}O_2$
147	Uridine	14.6617	244	$C_9H_{12}N_2O_6$
148	5-Methoxytryptamine	14.7032	190	$C_{11}H_{14}N_2O$
149	Androsterone	15.1675	290	$C_{19}H_{30}O_2$
150	1-Monopalmitin	15.1847	330	$C_{19}H_{38}O_4$
151	Sucrose	15.3673	342	$C_{12}H_{22}O_{11}$
152	Behenic acid	15.4738	340	$C_{22}H_{44}O_2$
153	Prostaglandin A2	15.4900	334	$C_{20}H_{30}O_4$
154	Xanthosine	15.5545	284	$C_{10}H_{12}N_4O_6$
155	7,8-Dimethylalloxazine	15.5709	242	$C_{12}H_{10}N_4O_2$
156	Lactose	15.6318	342	$C_{12}H_{22}O_{11}$
157	Cellobiose	15.9393	342	$C_{12}H_{22}O_{11}$
158	Prostaglandin E2	16.0093	363	$C_{20}H_{32}O_5$
159	7-Hydroxy-4-androstene-3,17-dione	16.1115	302	$C_{19}H_{26}O_3$

160	Monostearin	16.1220	358	$C_{21}H_{42}O_4$
161	Sophorose	16.1440	342	$C_{12}H_{22}O_{11}$
162	15-Keto-prostaglandin F2alpha	16.1534	352	$C_{20}H_{32}O_5$
163	Gentiobiose	16.3075	342	$C_{12}H_{22}O_{11}$
164	Dihydroquercetin	16.8704	304	$C_{15}H_{12}O_7$
165	7-alpha-Hydroxycholesterol	17.7250	402	$C_{27}H_{46}O_2$
166	5-Dihydrocortisone	17.7322	362	$C_{21}H_{30}O_5$

Supporting Table S3 Metabolites in urine detected by GC-MS/TOF

No	Name	R.T.	MW	MF
1	2-hydroxypyridine	8.9103	95	C_5H_5NO
2	Pyruvic acid	9.0134	88	$C_3H_4O_3$
3	Lactic acid	9.1416	90	$C_3H_6O_3$
4	Glycolic acid	9.3792	76	$C_2H_4O_3$
5	Alanine	9.7815	89	$C_3H_7NO_2$
6	Maleimide	9.8369	97	$C_4H_3NO_2$
7	Hydroxylamine	10.0161	33	H_3NO
8	2-Hydroxybutanoic acid	10.1025	104	$C_4H_8O_3$
9	Oxalic acid	10.1559	90	$C_2H_2O_4$
10	Sarcosine	10.2843	89	$C_3H_7NO_2$
11	2-Furoic Acid	10.3802	112	$C_5H_4O_3$
12	3-Hydroxypropanoate	10.3364	90	$C_3H_6O_3$
13	3-Hydroxypyridine	10.5112	95	C_5H_5NO
14	5-Aminovaleric acid lactam	10.4941	99	C_5H_9NO
15	3-Hydroxybutyric acid	10.5830	104	$C_4H_8O_3$
16	p-Cresol	10.6352	108	C_7H_8O
17	Sulfuric acid	10.7867	98	H_2O_4S
18	Lactamide	10.7372	89	$C_3H_7NO_2$
19	N-Methyl-DL-alanine	10.7801	103	$C_4H_9NO_2$
20	2-Amino-2-methylpropane-1,3-diol	10.8447	105	$C_4H_{11}NO_2$
21	Methyl Phosphate	10.9218	112	CH_5O_4P
22	Malonic acid	11.2335	104	$C_3H_4O_4$
23	2-Hydroxyvaleric acid	11.3225	118	$C_5H_{10}O_3$
24	Methylmalonic acid	11.3808	118	$C_4H_6O_4$
25	Valine	11.4089	117	$C_5H_{11}NO_2$
26	1-Aminocyclopropanecarb oxylic acid	11.4726	101	$C_4H_7NO_2$
27	Hydroxyurea	11.6781	76	$CH_4N_2O_2$
28	4-Hydroxybutyrate	11.8070	103	$C_4H_7O_3$-
29	2-Ketoadipate	11.8690	160	$C_6H_8O_5$
30	Dihydroxyacetone	11.9465	90	$C_3H_6O_3$
31	Benzoic acid	12.0081	122	$C_7H_6O_2$
32	Ethanolamine	12.1553	61	C_2H_7NO
33	Phosphate	12.1990	95	O_4P^{-3}
34	4-Vinylphenol	12.3251	120	C_8H_8O
35	Isoleucine	12.6073	131	$C_6H_{13}NO_2$
36	Maleic acid	12.6536	116	$C_4H_4O_4$
37	Phenylacetic acid	12.7614	136	$C_8H_8O_2$
38	Glycine	12.7818	75	$C_2H_5NO_2$
39	Succinate	12.8294	118	$C_4H_6O_4$

40	2,3-Dihydroxypyridine	12.8915	111	$C_5H_5NO_2$
41	Catechol	12.9529	110	$C_6H_6O_2$
42	2,2-Dimethylsuccinic Acid	12.9760	146	$C_6H_{10}O_4$
43	D-Glycerate	12.9858	106	$C_3H_6O_4$
44	Oxamide	13.0377	88	$C_2H_4N_2O_2$
45	1-Methylhydantoin	13.0797	114	$C_4H_6N_2O_2$
46	Picolinic acid	13.2024	123	$C_6H_5NO_2$
47	Uracil	13.1580	112	$C_4H_4N_2O_2$
48	Fumaric acid	13.2950	116	$C_4H_4O_4$
49	Allylmalonic acid	13.3042	144	$C_6H_8O_4$
50	Serine	13.3890	105	$C_3H_7NO_3$
51	3-Hydroxynorvaline	13.4145	133	$C_5H_{11}NO_3$
52	3-Cyanoalanine	13.5259	114	$C_4H_6N_2O_2$
53	Cycloleucine	13.5590	129	$C_6H_{11}NO_2$
54	3-Methylamino-1,2-propanediol	13.6788	105	$C_4H_{11}NO_2$
55	Tartronic acid	13.7293	120	$C_3H_4O_5$
56	Resorcinol	13.7681	110	$C_6H_6O_2$
57	O-acetylserine	13.9572	147	$C_5H_9NO_4$
58	Thymine	13.9815	126	$C_5H_6N_2O_2$
59	Glutaric Acid	14.0162	132	$C_5H_8O_4$
60	3-Aminopropionitrile	14.0374	70	$C_3H_6N_2$
61	2-Methylglutaric Acid	14.1495	146	$C_6H_{10}O_4$
62	3-Methylglutaric Acid	14.2742	146	$C_6H_{10}O_4$
63	beta-Alanine	14.3333	89	$C_3H_7NO_2$
64	Hydrocinnamic acid	14.3677	150	$C_9H_{10}O_2$
65	2-Aminophenol	14.4285	109	C_6H_7NO
66	Citraconic acid	14.4681	130	$C_5H_6O_4$
67	N-Ethylmaleamic acid	14.4823	143	$C_6H_9NO_3$
68	D-Erythronolactone	14.5380	118	$C_4H_6O_4$
69	L-Homoserine	14.5688	119	$C_4H_9NO_3$
70	2,4-Diaminobutyric acid	14.6006	118	$C_4H_{10}N_2O_2$
71	Erythrose	14.6432	158	$C_7H_{10}O_4$
72	3-Aminoisobutyric acid	14.7568	103	$C_4H_9NO_2$
73	2’-Hydroxyacetophenone	14.8031	136	$C_8H_8O_2$
74	Aminomalonic acid	14.8367	119	$C_3H_5NO_4$
75	Citramalic acid	14.8660	148	$C_5H_8O_5$
76	N-Methyl-L-glutamic acid	14.9224	197	$C_6H_{12}ClNO_4$
77	Maleamate	15.0190	115	$C_4H_5NO_3$
78	(S)-Malate	15.0367	134	$C_4H_6O_5$
79	Bis(2-hydroxypropyl)amine	15.1980	133	$C_6H_{15}NO_2$
80	Threitol	15.2613	124	$C_4H_{10}O_4$
81	Adipic acid	15.3081	146	$C_6H_{10}O_4$
82	Salicylate	15.4291	138	$C_7H_6O_3$

83	2-Hydroxyquinoline	15.4544	145	C_9H_7NO
84	Aspartic acid	15.4549	133	$C_4H_7NO_4$
85	Methionine	15.5073	149	$C_5H_{11}NO_2S$
86	trans-4-Hydroxy-L-proline	15.5527	131	$C_5H_9NO_3$
87	Acetol	15.5737	74	$C_3H_6O_2$
88	Oxoproline	15.5813	129	$C_5H_7NO_3$
89	4-Aminobutyric acid	15.6359	103	$C_4H_9NO_2$
90	Cumic Acid	15.7111	164	$C_{10}H_{12}O_2$
91	Threonic acid	15.8857	136	$C_4H_8O_5$
92	L-cysteine	15.9156	121	$C_3H_7NO_2S$
93	Creatine	15.9548	131	$C_4H_9N_3O_2$
94	3-Hydroxybenzoic acid	16.0980	90	$C_3H_6O_3$
95	2-Hydroxy-3-isopropylbutanedioic acid	16.1156	270	$C_9H_{18}O_5$
96	2-Oxoglutarate	16.1333	146	$C_5H_6O_5$
97	4-Hydroxyphenylethanol	16.1708	138	$C_8H_{10}O_2$
98	6-Hydroxynicotinic acid	16.1829	139	$C_6H_5NO_3$
99	Glycocyamine	16.1997	117	$C_3H_7N_3O_2$
100	Digitoxose	16.3875	148	$C_6H_{12}O_4$
101	D-Alanyl-D-alanine	16.4161	160	$C_6H_{12}N_2O_3$
102	threo-beta-Hyrdoxyaspartate	16.4548	149	$C_4H_7NO_5$
103	Pimelic acid	16.4919	160	$C_7H_{12}O_4$
104	beta-Glutamic acid	16.5167	118	$C_5H_9NO_4$
105	3-HydroxyphenylAcetic acid	16.5955	152	$C_8H_8O_3$
106	Glutamic acid	16.6472	147	$C_5H_9NO_4$
107	Asparagine	16.6528	132	$C_4H_8N_2O_3$
108	Tartaric acid	16.8014	150	$C_4H_6O_6$
109	Phenylalanine	16.8240	165	$C_9H_{11}NO_2$
110	4-Hydroxybenzoic acid	16.8520	138	$C_7H_6O_3$
111	4-Hydroxyphenylacetic acid	16.9620	152	$C_8H_8O_3$
112	Xylose	16.9920	152	$C_5H_{12}O_5$
113	Allose	17.0436	180	$C_6H_{12}O_6$
114	N-Acetyl-L-aspartic acid	17.1368	175	$C_6H_9NO_5$
115	1,2-Cyclohexanedione	17.2141	112	$C_6H_8O_2$
116	Ribose	17.2679	150	$C_5H_{10}O_5$
117	Taurine	17.3045	125	$C_2H_7NO_3S$
118	Ribonic acid, gamma-lactone	17.4023	148	$C_5H_8O_5$
119	3,6-Anhydro-D-galactose	17.5674	162	$C_6H_{10}O_5$
120	Levoglucosan	17.5771	162	$C_6H_{10}O_5$
121	Xylitol	17.5926	152	$C_5H_{12}O_5$
122	D-Arabitol	17.7646	152	$C_5H_{12}O_5$
123	6-Deoxy-D-glucose	17.8460	164	$C_6H_{12}O_5$
124	3-(3-hydroxyphenyl)propionic acid	17.9692	166	$C_9H_{10}O_3$

125	Fucose	18.0100	164	$C_6H_{12}O_5$
126	Orotic acid	18.0629	156	$C_5H_4N_2O_4$
127	2-Amino-1-phenylethanol	18.0735	137	$C_8H_{11}NO$
128	Sn-Glycerol 3-phosphate	18.2183	172	$C_3H_9O_6P$
129	Glucose-1-phosphate	18.2880	276	$C_6H_{13}O_{10}P$
130	Biuret	18.3173	103	$C_2H_5N_3O_2$
131	4-Hydroxy-3-methoxybenzoic acid	18.3567	168	$C_8H_8O_4$
132	2-Deoxy-D-glucose	18.3876	164	$C_6H_{12}O_5$
133	Gentisic acid	18.5007	154	$C_7H_6O_4$
134	Dehydroshikimic acid	18.5237	172	$C_7H_8O_5$
135	N-Acetyl-L-glutamic acid	18.5499	189	$C_7H_{11}NO_5$
136	5-Aminoimidazole-4-carboxamide	18.6399	126	$C_4H_6N_4O$
137	3-phosphoglycerate	18.7012	186	$C_3H_7O_7P$
138	Shikimic acid	18.7335	174	$C_7H_{10}O_5$
139	alpha-D-glucosamine 1-phosphate	18.7880	259	$C_6H_{14}NO_8P$
140	Isocitric acid	18.8326	192	$C_6H_8O_7$
141	2,8-Dihydroxyquinoline	19.0210	161	$C_9H_7NO_2$
142	1,5-Anhydroglucitol	19.1350	164	$C_6H_{12}O_5$
143	4-Aminobenzoic acid	19.1582	137	$C_7H_7NO_2$
144	beta-Mannosylglycerate	19.1855	270	$C_9H_{18}O_9$
145	Methyl-beta-D-galactopyranoside	19.2422	194	$C_7H_{14}O_6$
146	Quinic acid	19.2548	192	$C_7H_{12}O_6$
147	3-Hydroxyanthranilic acid	19.3938	153	$C_7H_7NO_3$
148	3,4-Dihydroxyphenylglycol	19.4224	170	$C_8H_{10}O_4$
149	Mannose	19.4463	180	$C_6H_{12}O_6$
150	Sorbose	19.4626	180	$C_6H_{14}O_6$
151	Allantoic acid	19.5466	176	$C_4H_8N_4O_4$
152	Gluconic lactone	19.5766	178	$C_6H_{10}O_6$
153	Galactose	19.5815	180	$C_6H_{12}O_6$
154	Glucose	19.6415	180	$C_6H_{12}O_6$
155	dl-p-Hydroxyphenyllactic acid	19.7179	182	$C_9H_{10}O_4$
156	D-Talose	19.8242	180	$C_6H_{12}O_6$
157	Lysine	19.9410	146	$C_6H_{14}N_2O_2$
158	3,4-Dihydroxymandelic acid	20.0066	184	$C_8H_8O_5$
159	Glucuronic acid	20.0315	194	$C_6H_{10}O_7$
160	Sorbitol	20.0555	182	$C_6H_{14}O_6$
161	4-Pyridoxic acid	20.1762	183	$C_8H_9NO_4$
162	Ascorbate	20.1860	176	$C_6H_8O_6$
163	Conduritol b epoxide	20.2620	162	$C_6H_{10}O_5$
164	D-Galacturonic acid	20.3450	194	$C_6H_{10}O_7$
165	Indole-3-acetic acid	20.4576	175	$C_{10}H_9NO_2$
166	N-alpha-Acetyl-L-ornithine	20.5421	174	$C_7H_{14}N_2O_3$
167	Galactonic acid	20.6390	196	$C_6H_{12}O_7$

168	Gluconic acid	20.7098	196	$C_6H_{12}O_7$
169	Pantothenic acid	20.7518	219	$C_9H_{17}NO_5$
170	Saccharic acid	20.9546	210	$C_6H_{10}O_8$
171	Glucosaminic acid	20.9775	195	$C_6H_{13}NO_6$
172	Palmitic acid	21.1859	256	$C_{16}H_{32}O_2$
173	L-Dopa	21.2950	197	$C_9H_{11}NO_4$
174	N-Acetyl-beta-D-mannosamine 1	21.3266	221	$C_8H_{15}NO_6$
175	N-Acetyl-D-galactosamine 1	21.4259	221	$C_8H_{15}NO_6$
176	4-oxo-1H-Quinoline-2-carboxylic acid	21.4980	189	$C_{10}H_7NO_3$
177	myo-Inositol	21.5809	180	$C_6H_{12}O_6$
178	Uric acid	21.6224	168	$C_5H_4N_4O_3$
179	Ferulic acid	21.6832	194	$C_{10}H_{10}O_4$
180	Flavanone	22.0211	224	$C_{15}H_{12}O_2$
181	Noradrenaline	22.1101	319	$C_{12}H_{17}NO_9$
182	d-Glucoheptose	22.1859	210	$C_7H_{14}O_7$
183	Glutathione	22.2165	612	$C_{20}H_{32}N_6O_{12}S_2$
184	Indolelactate	22.3877	205	$C_{11}H_{11}NO_3$
185	5-Methoxyindole-3-acetic acid	22.4193	205	$C_{11}H_{11}NO_3$
186	Linoleate	22.7176	280	$C_{18}H_{32}O_2$
187	5-Hydroxyindole-3-acetic acid	22.7166	191	$C_{10}H_9NO_3$
188	Stearic acid	22.9682	284	$C_{18}H_{36}O_2$
189	Xanthurenic acid	23.1332	205	$C_{10}H_7NO_4$
190	Glucoheptonic acid	23.1599	226	$C_7H_{14}O_8$
191	Fructose-6-phosphate	23.4201	260	$C_6H_{13}O_9P$
192	Phenyl beta-D-glucopyranoside	23.5474	256	$C_{12}H_{16}O_6$
193	Glucose-6-phosphate	23.6278	260	$C_6H_{13}O_9P$
194	Melatonin	24.3296	232	$C_{13}H_{16}N_2O_2$
195	6-Phosphogluconic acid	24.3573	276	$C_6H_{13}O_{10}P$
196	D-Erythro-sphingosine	24.5117	299.5	$C_{18}H_{37}NO_2$
197	Cytidine-monophosphate degr prod	24.7431	-	-
198	5-Methoxytryptamine	24.7807	190	$C_{11}H_{14}N_2O$
199	Salicin	24.9550	286	$C_{13}H_{18}O_7$
200	2-Monopalmitin	25.5282	330	$C_{19}H_{38}O_4$
201	Arbutin	25.6472	272	$C_{12}H_{16}O_7$
202	Inosine	25.7107	268	$C_{10}H_{12}N_4O_5$
203	Sucrose	26.0175	342	$C_{12}H_{22}O_{11}$
204	Lactose	26.3221	342	$C_{12}H_{22}O_{11}$
205	Cellobiose	26.5916	342	$C_{12}H_{22}O_{11}$
206	Resveratrol	26.7610	228	$C_{14}H_{12}O_3$
207	Trehalose	26.8475	342	$C_{12}H_{22}O_{11}$
208	Lactobionic Acid	26.8842	358	$C_{12}H_{22}O_{12}$
209	Prostaglandin E2	26.9876	363	$C_{20}H_{32}O_5$

210	Maltose	27.0350	342	$C_{12}H_{22}O_{11}$
211	Guanosine	27.0210	283	$C_{10}H_{13}N_5O_5$
212	Chrysin	27.1546	254	$C_{15}H_{10}O_4$
213	2'-Deoxyguanosine	27.2610	267	$C_{10}H_{13}N_5O_4$
214	Gentiobiose	27.5038	342	$C_{12}H_{22}O_{11}$
215	Lignoceric acid	27.5518	368	$C_{24}H_{48}O_2$
216	Piceatannol	27.7418	244	$C_{14}H_{12}O_4$
217	Loganin	28.0419	390	$C_{17}H_{26}O_{10}$
218	Melibiose	28.0091	360	$C_{12}H_{24}O_{12}$
219	Dihydroquercetin	28.2019	304	$C_{15}H_{12}O_7$
220	Naringenin	28.4984	272	$C_{15}H_{12}O_5$
221	daidzein	28.5383	254	$C_{15}H_{10}O_4$
222	Galactinol	28.6305	342	$C_{12}H_{22}O_{11}$
223	Cholecalciferol	28.8119	384	$C_{27}H_{44}O$
224	Tetrahydrocorticosterone	28.8573	350	$C_{21}H_{34}O_4$
225	Cerotinic acid	28.9491	396	$C_{26}H_{52}O_2$
226	21-Hydroxypregnenolone	29.0407	332	$C_{21}H_{32}O_3$
227	2'-Deoxyadenosine 5'-monophosphate	29.3147	331	$C_{10}H_{14}N_5O_6P$
228	Chlorogenic Acid	29.3983	354	$C_{16}H_{18}O_9$
229	Cortexolone	29.5777	346	$C_{21}H_{30}O_4$
230	Trehalose-6-phosphate	29.7176	422	$C_{12}H_{23}O_{14}P$

Supporting Table S4 The information of selected metabolites in serum samples for 50 days

No	Peak	R.T.	MW	MF	↑or↓
1	Xylitol	11.0910	152	$C_5H_{12}O_5$	↓
2	Phosphate	8.30157	95	O_4P^{-3}	↑
3	beta-D-Fructose 2,6-bisphosphate	13.5749	340	$C_6H_{14}O_{12}P_2$	↓
4	D-Arabitol	11.1201	152	$C_5H_{12}O_5$	↓
5	2-Hydroxyvaleric acid	7.85292	118	$C_5H_{10}O_3$	↓
6	Toluenesulfonic acid	10.6794	172	$C_7H_8O_3S$	↓
7	3-Hydroxy-3-methylglutaric acid	10.4500	162	$C_6H_{10}O_5$	↓
8	Biuret	11.4195	103	$C_2H_5N_3O_2$	↓
9	Uracil-5-carboxylic acid	11.44290	156	$C_5H_4N_2O_4$	↑
10	Guanidinosuccinic acid	11.03110	175	$C_5H_9N_3O_4$	↓

Supporting Table S5 The information of selected metabolites in urine samples for 20, 40, 50 days

No	Peak	R.T.	MW	MF	↑or↓		
					20	40	50
1	Sorbitol	20.0555	182	$C_6H_{14}O_6$	↑	↑	-
2	Galactose	19.5815	180	$C_6H_{12}O_6$	↑	↑	-
3	3-Phosphoglycerate	18.7012	186	$C_3H_7O_7P$	↑	↑	-
4	3-(3-Hydroxyphenyl)propionic acid	17.9692	166	$C_9H_{10}O_3$	↓	-	-
5	2-Oxoglutarate	16.1333	146	$C_5H_6O_5$	↑	↑	-
6	Threonic acid	15.8857	136	$C_4H_8O_5$	↑	↑	-
7	Allose	17.0436	180	$C_6H_{12}O_6$	↑	↑	↑
8	N-Acetyl-L-glutamic acid	18.5499	189	$C_7H_{11}NO_5$	↑	-	-
9	Saccharic acid	20.9546	210	$C_6H_{10}O_8$	↑	-	-
10	Linoleate	22.7176	280	$C_{18}H_{32}O_2$	↓	-	-
11	Arbutin	25.6472	272	$C_{12}H_{16}O_7$	↑	-	-
12	beta-D-Fructose 2,6-bisphosphate	22.4053	340	$C_6H_{14}O_{12}P_2$	↑	-	-
13	Mannose	19.4463	180	$C_6H_{12}O_6$	↑	-	-
14	Glutathione	22.2165	612	$C_{20}H_{32}N_6O_{12}S_2$	↓	-	-
15	Glucosaminic acid	20.9775	195	$C_6H_{13}NO_6$	↓	-	-
16	2'-Hydroxyacetophenone	14.8031	136	$C_8H_8O_2$	↑	-	-
17	Benzoic acid	12.0081	122	$C_7H_6O_2$	-	↑	-
18	Oxalic acid	10.1559	90	$C_2H_2O_4$	-	↓	-
19	Quinic acid	19.2548	192	$C_7H_{12}O_6$	-	↑	-
20	Hydroxyurea	11.6781	76	$CH_4N_2O_2$	-	↓	-
21	Isoleucine	12.6073	131	$C_6H_{13}NO_2$	-	↑	-
22	Levoglucosan	17.5771	162	$C_6H_{10}O_5$	-	↑	-
23	1,2,4-Benzenetriol	16.4659	126	$C_6H_6O_3$	-	↓	-
24	4-Hydroxyphenylethanol	16.1708	138	$C_8H_{10}O_2$	-	-	↓
25	Lactose	26.3221	342	$C_{12}H_{22}O_{11}$	-	-	↑
26	Allylmalonic acid	13.3042	144	$C_6H_8O_4$	-	-	↑
27	Dihydroxyacetone	11.9465	90	$C_3H_6O_3$	-	-	↓

Supporting Table S6 The information of selected metabolites for 80 days

No	Peak	R.T.	MW	MF	↑or↓	
					Serum	Urine
1	Creatine	10.2464	131	$C_4H_9N_3O_2$	↓	-
2	D-Glycerate	8.67022	106	$C_3H_6O_4$	↑	-
3	beta-Alanine	9.40244	89	$C_3H_7NO_2$	↑	-
4	Phosphate	8.30157	95	O_4P^{-3}	↑	-
5	Palmitic acid	12.8631	256	$C_{16}H_{32}O_2$	↑	-
6	Hydroxylamine	7.19154	33	H_3NO	↑	-
7	beta-Glutamic acid	10.5416	147	$C_5H_9NO_4$	↑	-
8	1,5-Anhydroglucitol	11.9496	164	$C_6H_{12}O_5$	↑	-
9	Pyrrole-2-Carboxylic Acid	8.94082	111	$C_5H_5NO_2$	↑	-
10	Guanidinosuccinic acid	11.0311	175	$C_5H_9N_3O_4$	↓	-
11	Glutamine	10.1662	146	$C_5H_{10}N_2O_3$	↑	-
12	2-Monopalmitin	25.5282	330	$C_{19}H_{38}O_4$	-	↑

Supporting Table S7 The information of selected metabolites for the changes of serum samples in 80 days [a]

No	Peak	R.T.	MW	MF	↑or↓			
					20	40	50	80
1	Glycine	8.5798,0	75	$C_2H_5NO_2$	↓	-	-	-
2	Fructose	11.915,0	180	$C_6H_{12}O_6$	↑	-	↑	-
3	sn-Glycerol 3-phosphate	11.3271,0	172	$C_3H_9O_6P$	↑	-	↑	-
4	Hydroxylamine	7.19154,0	33	H_3NO	↓	-	-	-
5	2-Oxoglutarate	10.2971,0	146	$C_5H_6O_5$	↓	↓	-	-
6	Lactic acid	6.70711,0	90	$C_3H_6O_3$	↓	-	-	-
7	Pantothenic acid	12.5837,0	219	$C_9H_{17}NO_5$	↓	-	-	-
8	Fumaric acid	8.86047,0	116	$C_4H_4O_4$	↓	-	-	-
9	Pyruvic acid	6.6365	88	$C_3H_4O_3$	↓	-	↓	-
10	Pipecolinic acid	9.02289,0	129	$C_6H_{11}NO_2$	↓	↓	↓	-
11	Erythrose	9.47857,0	158	$C_7H_{10}O_4$	↓	-	-	-
12	Glucose-1-phosphate	11.3696,0	276	$C_6H_{13}O_{10}P$	↓	-	↓	↓
13	3-Aminoisobutyric acid	9.63532,0	103	$C_4H_9NO_2$	↓	-	-	-
14	Linoleic acid methyl ester	13.1818,0	294	$C_{19}H_{34}O_2$	↑	-	↑	↑
15	3-Hydroxy-3-methylglutaric acid	10.45,0	162	$C_6H_{10}O_5$	↓	-	↓	-
16	Quinic acid	11.8627,0	192	$C_7H_{12}O_6$	↑	↑	↑	↑
17	Dihydroquercetin	16.8704,0	304	$C_{15}H_{12}O_7$	↑	-	-	-
18	Cytosin	10.7312,0	111	$C_4H_5N_3O$	↓	-	-	-
19	Citramalic acid	9.73983,0	148	$C_5H_8O_5$	↓	-	-	-
20	Asparagine	10.881,0	132	$C_4H_8N_2O_3$	-	↑	-	↑
21	Glucose-6-phosphate	14.0119,0	260	$C_6H_{13}O_9P$	-	↑	↑	↑
22	Gentiobiose	16.3075,0	342	$C_{12}H_{22}O_{11}$	-	↓	-	-
23	Allose	10.8388,0	180	$C_6H_{12}O_6$	-	↑	-	-
24	D-erythronolactone	10.4997,0	118	$C_4H_6O_4$	↓	↓	↓	↓
25	3-Phenyllactic acid	10.4304,0	166	$C_9H_{10}O_3$	-	↑	-	↓
26	3-Hydroxynorvaline	9.0679,0	133	$C_5H_{11}NO_3$	-	↑	-	↑
27	Pyrrole-2-Carboxylic Acid	8.94082,0	111	$C_5H_5NO_2$	-	↑	↑	↑
28	myo-Inositol	13.0599,0	180	$C_6H_{12}O_6$	-	-	↑	↑
29	Aspartic acid	9.94418,0	133	$C_4H_7NO_4$	-	-	↑	-
30	Tryptophan	13.6964,0	204	$C_{11}H_{12}N_2O_2$	-	-	↓	-
31	Succinate	8.60869,0	118	$C_4H_6O_4$	-	-	↑	-
32	Thymidine	8.35004,0	242	$C_{10}H_{14}N_2O_5$	-	-	↓	-
33	Toluenesulfonic acid	10.6794,0	172	$C_7H_8O_3S$	-	-	↓	-
34	Cellobiose	15.9393,0	342	$C_{12}H_{22}O_{11}$	-	-	↓	-

35	(S)-Malate	9.72681,0	134	$C_4H_6O_5$	-	-	↑	↑
36	Linolenic acid	13.6762,0	278	$C_{18}H_{30}O_2$	-	-	↓	-
37	2-Deoxy-D-galactose	11.5702,0	164	$C_6H_{12}O_5$	-	-	↓	-
38	Acetol	11.0419,0	74	$C_3H_6O_2$	-	-	↓	-
39	N-Acetylserotonin	14.5631,0	218	$C_{12}H_{14}N_2O_2$	-	-	↑	-
40	7-Hydroxy-4-androstene-3,17-dione	16.1115,0	302	$C_{19}H_{26}O_3$	-	-	↑	-
41	Isoleucine	8.45948,0	131	$C_6H_{13}NO_2$	-	-	-	↑
42	Taurine	10.9317,0	125	$C_2H_7NO_3S$	-	-	-	↑
43	beta-Alanine	9.40244,0	89	$C_3H_7NO_2$	-	-	-	↑
44	Phosphate	8.30157,0	95	O_4P^{-3}	-	-	-	↑
45	L-Cysteine	10.1999,0	121	$C_3H_7NO_2S$	-	-	-	↑
46	beta-Hydroxymyristic acid	12.7362,0	244	$C_{14}H_{28}O_3$	-	-	-	↑
47	7,8-Dimethylalloxazine	15.5709,0	242	$C_{12}H_{10}N_4O_2$	-	-	-	↑
48	3-Hydroxypropanoate	10.27,0	90	$C_3H_6O_3$	-	-	-	↑
49	N-Acetyl-beta-D-mannosamine	13.1442,0	221	$C_8H_{15}NO_6$	-	-	-	↑
50	Methionine sulfoxide	11.506,0	181	$C_5H_{11}NO_4S$	-	-	-	↑

a The difference metabolites of control group have been eliminated.

Supporting Table S8 The information of selected metabolites for the changes of urine samples in 80 days [a]

No	Peak	R.T.	MW	MF	↑or↓			
					20	40	50	80
1	Glutaric Acid	14.0162,0	132	$C_5H_8O_4$	↓	-	-	-
2	daidzein	28.5383,0	254	$C_{15}H_{10}O_4$	↓	-	-	-
3	Lactamide	10.7372,0	89	$C_3H_7NO_2$	↓	↓	-	↓
4	2-Amino-1-phenylethanol	18.0735,0	137	$C_8H_{11}NO$	↓	↓	↓	↓
5	Threonic acid	15.8857,0	136	$C_4H_8O_5$	↑	↑	-	-
6	Salicylate	15.4291,0	138	$C_7H_6O_3$	↓	↓	↓	
7	Glucoheptonic acid	23.1599,0	226	$C_7H_{14}O_8$	↓	-	-	-
8	Chlorogenic Acid	29.3983,0	354	$C_{16}H_{18}O_9$	↓	↓	-	-
9	3,4-Dihydroxymandelic acid	20.0066,0	184	$C_8H_8O_5$	↓	-	-	-
10	1-Aminocyclopropanecarboxylic acid	11.4726,0	101	$C_4H_7NO_2$	↑	-	-	-
11	Dehydroshikimic acid	18.5237,0	172	$C_7H_8O_5$	↓	-	↓	-
12	Bis(2-hydroxypropyl)amine	15.198,0	133	$C_6H_{15}NO_2$	↓	-	-	-
13	N-ethylmaleamic acid	14.4823,0	143	$C_6H_9NO_3$	↓	↓	↓	-
14	Glucose	19.6415,0	198	$C_6H_{14}O_7$	-	↓	-	↓
15	Ribose	17.2679,0	150	$C_5H_{10}O_5$	-	↓	↓	-
16	6-Deoxy-D-glucose	17.846,0	164	$C_6H_{12}O_5$	-	↓	↓	-
17	1-Methylhydantoin	13.0797,0	114	$C_4H_6N_2O_2$	-	↓	-	-
18	Oxalic acid	10.1559,0	94	$C_2H_2O_4$	-	↓	-	-
19	Fucose	18.01,0	164	$C_6H_{12}O_5$	-	↓	↓	-
20	Sorbose	19.4626,0	180	$C_6H_{12}O_6$	-	↑	↑	-
21	Gentiobiose	27.5038,0	342	$C_{12}H_{22}O_{11}$	-	↑	-	-
22	Quinic acid	19.2548,0	192	$C_7H_{12}O_6$	-	↑	-	-
23	Indolelactate	22.3877,0	205	$C_{11}H_{11}NO_3$	-	↓	↓	-
24	Phosphate	12.199,0	95	O_4P^{-3}	-	↓	↓	↓
25	Glutamic acid	16.6472,0	147	$C_5H_9NO_4$	-	↓	↓	↓
26	Allose	17.0436,0	180	$C_6H_{12}O_6$	-	↓	-	↓
27	4-Hydroxybutyrate	11.807,0	103	$C_4H_7O_3$-	-	↓	-	-
28	Pantothenic acid	20.7518,0	219	$C_9H_{17}NO_5$	-	↓	-	↓
29	Prostaglandin E2	26.9876,0	352	$C_{20}H_{32}O_5$	-	↓	-	-
30	Cumic Acid	15.7111,0	164	$C_{10}H_{12}O_2$	-	↓	↓	-
31	Glucosaminic acid	20.9775,0	195	$C_6H_{13}NO_6$	-	↓	-	-
32	Loganin	28.0419,0	390	$C_{17}H_{26}O_{10}$	-	↓	-	-

33	5-Aminoimidazole-4-carboxamide	18.6399,0	126	$C_4H_6N_4O$	-	↓	-	-
34	Sorbitol	20.0555,0	182	$C_6H_{14}O_6$	-	-	↓	↓
35	N-Methyl-L-glutamic acid	14.9224,0	161	$C_6H_{11}NO_4$	-	-	↓	-
36	Ascorbate	20.186,0	176	$C_6H_8O_6$	-	-	↓	-
37	N-Acetyl-L-glutamic acid	18.5499,0	189	$C_7H_{11}NO_5$	-	-	↓	-
38	threo-beta-Hyrdoxyaspartate	16.4548,0	149	$C_4H_7NO_5$	-	-	↓	-
39	Dihydroxyacetone	11.9465,0	90	$C_3H_6O_3$	-	-	↓	-
40	2-Monopalmitin	25.5282,0	330	$C_{19}H_{38}O_4$	-	-	↓	-
41	Glycine	12.7818,0	75	$C_2H_5NO_2$	-	-	-	↓
42	3-Hydroxypyridine	10.5112,0	95	C_5H_5NO	-	-	-	↓
43	Galactose	19.5815,0	180	$C_6H_{12}O_6$	-	-	-	↓
44	Hydroxylamine	10.0161,0	33	H_3NO	-	-	-	↓
45	Conduritol b epoxide	20.262,0	162	$C_6H_{10}O_5$	-	-	-	↓
46	Cycloleucine	13.559,0	129	$C_6H_{11}NO_2$	-	-	-	↓
47	2-Oxoglutarate	16.1333,0	146	$C_5H_6O_5$	-	-	-	↓
48	p-Cresol	10.6352,0	108	C_7H_8O	-	-	-	↓
49	dl-p-Hydroxyphenyllactic acid	19.7179,0	182	$C_9H_{10}O_4$	-	-	-	↓
50	Hydroxyurea	11.6781,0	76	$CH_4N_2O_2$	-	-	-	↓
51	Picolinic acid	13.2024,0	123	$C_6H_5NO_2$	-	-	-	↓
52	Chrysin	27.1546,0	254	$C_{15}H_{10}O_4$	-	-	-	↓
53	Arbutin	25.6472,0	272	$C_{12}H_{16}O_7$	-	-	-	↓
54	Levoglucosan	17.5771,0	162	$C_6H_{10}O_5$	-	-	-	↓
55	3-Cyanoalanine	13.5259,0	114	$C_4H_6N_2O_2$	-	-	-	↓
56	2-Amino-2-methylpropane-1,3-diol 2	10.8447,0	105	$C_4H_{11}NO_2$	-	-	-	↓
57	Trehalose	26.8475,0	342	$C_{12}H_{22}O_{11}$	-	-	-	↑

a The difference metabolites of control group have been eliminated.

Supporting Table S9 The pathway analysis data of 0-50 days

**Metabolic pathway**	**Serum Metabolites**						**Urine Metabolites**					
	***p* value**			**Impact factor**			***p* value**			**Impact factor**		
KWG vs Control												
	50			50			20	40	50	20	40	50
**Fructose and mannose metabolism**	0.9128			0			0.0003	0.1159	-	0.0314	0	-
**Galactose metabolism**	-			-			0.0165	0.1555	0.0547	0	0	0.016
**Glycerolipid metabolism**	-						0.1330	0.1109	0.0382	0	0	0
**Linoleic acid metabolism**	-			-			0.0387	-	-	1	-	-
**D-Glutamine and D-glutamate metabolism**	-			-			0.0387	0.0317	-	0	0	-
Dynamic perspective supplement (20, 40, 50 days vs 0 days)												
	20	40	50	20	40	50	20	40	50	20	40	50
**Citrate cycle (TCA cycle)**	0.0006	0.0422	0.0032	0.1667	0.0680	0.1427	-	-	-	-	-	-
**Butanoate metabolism**	0.0137	0.0422	0.0377	0	0	0	-	-	-	-	-	-
**Alanine, aspartate and glutamate metabolism**	0.0011	0.0505	0.0527	0.0665	0.0633	0	-	-	-	-	-	-
**Glycine, serine and threonine metabolism**	0.0337	-	0.0878	0.2919	-	0	-	-	-	-	-	-
**Pyruvate metabolism**	-	-	0.0449	-	-	0.1875	-	-	-	-	-	-
**Tryptophan metabolism**	-	-	0.1335	-	-	0.1982	-	-	-	-	-	-
**D-Glutamine and D-glutamate metabolism**	0.0456	0.0107	-	0	0	-	-	-	-	-	-	-

Supporting Table S10 The pathway analysis data of 50-80 days

Metabolic pathway	Serum Metabolites		Urine Metabolites	
	p value	Impact factor	*p* value	Impact factor
KWG vs Control				
**Glycine, serine and threonine metabolism**	0.0099	0	-	-
Dynamic perspective supplement (80 days vs 0 days)				
**beta-Alanine metabolism**	0.0106	0.4444	-	-
**Taurine and hypotaurine metabolism**	0.0018	0.4286	-	-
**Propanoate metabolism**	0.0177	0	-	-
**Pantothete and CoA biosynthesis**	0.0066	0	0.1216	0.0204
**Galactose metabolism**	0.2019	0	0.0195	0.0364
**D-Glutamine and D-glutamate metabolism**	-	-	0.0421	0

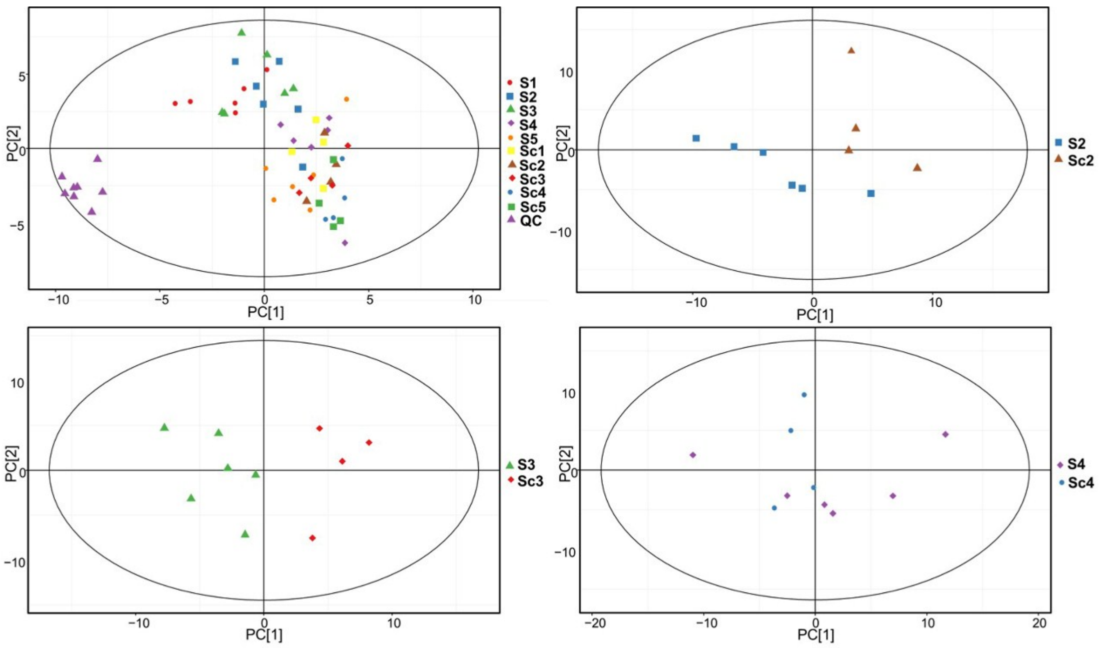

Supporting Figure. S4 Principal component analysis (PCA) score plots (Serum)

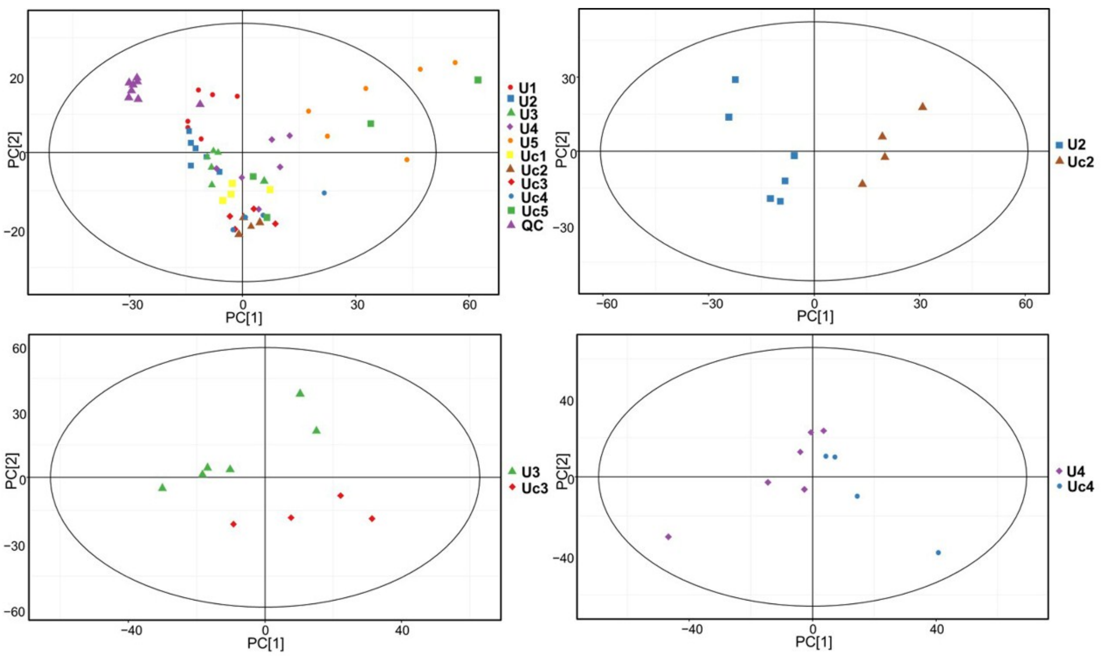

Supporting Figure. S5 Principal component analysis (PCA) score plots (Urine)
